# Plxdc2 Is a Mitogen for Neural Progenitors

**DOI:** 10.1371/journal.pone.0014565

**Published:** 2011-01-21

**Authors:** Suzanne F. C. Miller-Delaney, Ivo Lieberam, Paula Murphy, Kevin J. Mitchell

**Affiliations:** 1 Smurfit Institute of Genetics and Institute of Neuroscience, Trinity College Dublin, Dublin, Ireland; 2 Howard Hughes Medical Institute, Department of Biochemistry and Molecular Biophysics, Center for Neurobiology and Behavior, Columbia University, New York, New York, United States of America; 3 Department of Zoology, Trinity College Dublin, Dublin, Ireland; Universidade Federal do Rio de Janeiro, Brazil

## Abstract

The development of different brain regions involves the coordinated control of proliferation and cell fate specification along and across the neuraxis. Here, we identify Plxdc2 as a novel regulator of these processes, using in ovo electroporation and in vitro cultures of mammalian cells. Plxdc2 is a type I transmembrane protein with some homology to nidogen and to plexins. It is expressed in a highly discrete and dynamic pattern in the developing nervous system, with prominent expression in various patterning centres. In the chick neural tube, where Plxdc2 expression parallels that seen in the mouse, misexpression of Plxdc2 increases proliferation and alters patterns of neurogenesis, resulting in neural tube thickening at early stages. Expression of the Plxdc2 extracellular domain alone, which can be cleaved and shed in vivo, is sufficient for this activity, demonstrating a cell non-autonomous function. Induction of proliferation is also observed in cultured embryonic neuroepithelial cells (ENCs) derived from E9.5 mouse neural tube, which express a Plxdc2-binding activity. These experiments uncover a direct molecular activity of Plxdc2 in the control of proliferation, of relevance in understanding the role of this protein in various cancers, where its expression has been shown to be altered. They also implicate Plxdc2 as a novel component of the network of signalling molecules known to coordinate proliferation and differentiation in the developing nervous system.

## Introduction

Expression of diffusible molecules controlling both proliferation and cell fate specification from defined organising centres in the developing nervous system underlies the coordination of differentiation and growth of different brain regions. Many important families of secreted molecules involved in these processes have been identified, including members of the bone morphogenetic protein (BMP), fibroblast growth factor (Fgf), insulin-like growth factor (Igf) and Wnt families as well as Sonic Hedgehog (Shh). The midbrain-hindbrain boundary (MHB), which expresses Wnt1 and Fgf8, is one of several local signalling centres in the neuroepithelium which refines AP specification of the brain [1]. DV patterning is influenced by the floor plate, which expresses ventralising factors including sonic hedgehog (Shh) and nodal [2] and the roof plate at the dorsal midline, which expresses members of the BMP and Wnt families [3], [4]. Differential dorsal and ventral growth of the brain is also co-ordinated via a signalling cascade of Shh, FGF and Wnt activity [5], [6]. While advances have been made in understanding how growth and patterning are coordinated in the developing spinal cord [7] understanding of the coordination of these processes in the developing brain remains fragmented.

We have previously described the expression pattern of the transmembrane protein Plexin domain-containing 2 (Plxdc2) in the developing mouse embryo. This gene was isolated in a gene trap screen for novel transmembrane proteins (mouse line KST37) and was of particular interest based on its protein architecture and expression pattern [8], [9]. The mouse *Plxdc2* gene encodes a type I transmembrane protein of 530 amino acids, characterised by an extracellular region of weak nidogen homology and a plexin repeat or PSI domain, a domain found in several known axon guidance molecules. At late stages, Plxdc2 is expressed in restricted subsets of neurons and discrete brain nuclei [8], [9], suggesting a possible role in specification of neuronal connectivity. At mid-embryonic stages (E9.5–E11.5), however, Plxdc2 expression is found in patterning centres of the brain, including the cortical hem, midbrain–hindbrain boundary (MHB) and floorplate, indicating possible functions in earlier neurodevelopmental processes. Remarkable similarities between the expression of Plxdc2 and that of a number of members of the Wnt family (including Wnt1, Wnt3a, Wnt5a and Wnt 8b) have been noted, particularly in the regions of the cortical hem and midbrain-hindbrain boundary (MHB) [9]. Recent studies have shown that the extracellular portion of the Plxdc2 protein can be cleaved near the membrane, shedding the ectodomain [10], [11].

In the human, mouse and chick *Plxdc2* has one related gene, *Plxdc1*, which was isolated in a screen for genes upregulated in human colorectal tumour endothelium (and originally named *tumour endothelial marker 7*, *TEM7* [12], [13]. Plxdc1 is upregulated in the stromal endothelial cells of various tumours and has been shown to be upregulated and essential during endothelial cell capillary morphogenesis [12], [13], [14], [15]. No such functional requirement for Plxdc2 in this process has been demonstrated, although it is upregulated in stromal endothelial cells of various tumours, including human colorectal cancer and glioblastoma [12]. Expression of Plxdc2 is also altered in other cancers [16], [17] and in a number of conditions where a balance between proliferation, apoptosis and cellular arrest is involved [18], [19], [20]. Despite these correlative data suggesting the possible importance of Plxdc2 in these processes no cellular function has yet been ascribed to the protein.

In this study, we present evidence that Plxdc2 can act as a mitogen in the developing nervous system. Using in ovo electroporation techniques, we demonstrate that the overexpression and ectopic expression of Plxdc2 induces neural tube thickening in the chick embryo. This thickening is associated with an increase in proliferation and with alteration of the normal expression of the proneural marker Cash1. The extracellular domain alone is sufficient to induce this effect, indicating a cell non-autonomous function. We also show directly that treatment with the extracellular portion of the Plxdc2 protein increases proliferation in a embryonic neuroepithelial cell line cultured from the E9.5 murine neural tube. These findings implicate Plxdc2 as a novel component of the network of signaling molecules known to coordinate proliferation and differentiation in the developing nervous system.

## Methods

All animal procedures were performed in accordance with the European Communities Council Directive (86/609/EEC) and were reviewed and approved by the Trinity College BioResources Committee under license from the Department of Health, Dublin, Ireland (B100/3527).

### Plxdc2-eGFP knock-in

Mouse genomic fragments used to generate Plxdc2-eGFP knock-in mice were derived from C57B6 genomic BAC RP23–390M20. The DNA fragments serving as the 5′ arm of homology (7.8 kb) and the 3′ arm of homology (3.8 kb) were amplified by PCR (Expand Long Template PCR System, Roche), cloned into pCRXL-TOPO (Invitrogen) and sequenced. These fragments were then assembled in the vector pEZ-FRT-Lox-DT [21] such that the arms of homology flank the 5′splice substrate/eGFP/pA cassette [22] followed by the loxP-flanked neomycin resistance marker derived from pEasy-Flox [23]. The resulting modification to exon 1 eliminates the start codon and most of the signal peptide and is designed to express eGFP under the control of the *Plxdc2* promotor (see Supplemental Fig. S1). The linearized gene targeting construct was electroporated into C57B6-derived CMT2 ES cells (Cell & Molecular Technologies, Phillipsburg, NJ, USA), and homologous recombinants were identified by Southern Blot. Recombinant clones were injected into C57B6 blastocysts to generate chimeric founders that transmitted the mutant allele. The loxP-flanked neomycin selection marker was deleted in vivo using the EIIa:Cre deleter transgene [24].

RNA was extracted from the cerebella of adult wildtype, heterozygous and homozygous Plxdc2GFP mice using TRI Reagent (Sigma, as per protocol). Three primer pairs were designed to regions within exon 7 and exon 14 of the Plxdc2 transcript (downstream of the targeted insertion of the 5S-EGFP-pA cassette in exon 1 of Plxdc2). The most sensitive of these primer pairs amplified a product of 61 bp from exon 7 of the *Plxdc2* transcript and was subsequently used for calculation of Plxdc2 transcript levels (Forward primer 5′-CCAGTGAAAGTCGGGTTGTCTG-3′; reverse primer 5′-TGGGTATTTGCTGGATCCTGTG-3′).

### Chicken husbandry

Fertilised chicken eggs (Ross 500 strain) were obtained from Cobbs Hatchery, Kildare and incubated at 37.5°C with controlled humidity in a Solway 24 incubator (Solway Feeders Ltd.). Chick embryos were staged according to Hamburger and Hamilton (HH) morphological criteria [25].

### In situ hybridization

DIG-labelled RNA probes for chick *Plxdc2* were produced using ChEST853h24 (Geneservice) as a template. Chicken achaete-scute homolog-like 1 (Cash1) probes have previously been described [26] and were a kind gift of T.A. Reh (University of Washington, USA). Whole mount in situ hybridisation was carried out largely according to Miller et al., [9]. For Cash1 book preparations, neural tubes were dissected and cut along the ventral or dorsal midline prior to mounting on slides in 20% glycerol/PBS. Plxdc2 in situ hybridisation on free floating sections was carried out according to Riddle et al., [27] with minor adjustments. HH stage 36 brains were fixed in 4% PFA, overnight at 4°C. The hindbrain was embedded in 4% low melting point agarose (Sigma) and 100 µm free floating sections collected using a vibrating microtome (Leica, model VT1000S). Brain sections were incubated in probe overnight at 65°C in a sealed humidified chamber. Cash1 in situ hybridisation on cryostat sections of the chick was carried out according to Schaeren-Wiemers and Gerfin-Moser [28].

### Optical Projection Tomography (OPT)

In situ hybridised embryos were prepared for OPT as outlined in Miller et al., [9]. OPT was carried out as described [29] on a prototype scanner built at the MRC Human Genetics Unit Edinburgh (commercial version now available from BioOptonics). Software for 3D reconstruction, volume rendering and analysis was kindly provided by James Sharpe and the Edinburgh Mouse Atlas Project (EMAP) (MA3Dview and MAPaint downloadable from EMAGE, http://genex.hgu.mrc.ac.uk/emage/home.php). For neural tube thickening analysis, the thickness of virtual sections through the neural tube was measured using Cell A Software.

### Cloning of *Plxdc2*

RNA was extracted from HH stage 21 chick embryos using TRI Reagent (Sigma). Reverse transcription of RNA was carried out using Superscript II reverse transcriptase (Invitrogen) and Oligo d(T) primer (New England Biolabs). Primers were designed to chick *Plxdc2* cDNA sequence (Forward primer located prior to the start codon 5′-CGGAATTCCGGAGAGTTGTCTCGGCA-3′; Reverse primer located upstream to the stop codon 5′-CGCTCGAGTGCTCTGATACAATGAAGCC-3′). PCR was carried out using Phusion High-Fidelity DNA Polymerase (Finnzymes). A product of approximately 1.6 kb, corresponding to the predicted length of the *Plxdc2* transcript (1611 bp), was amplified and cloned into pcDNA3.1myc-His(B) plasmid at EcoRI and XhoI sites. The extracellular mouse *Plxdc2* plasmid (msPlxdc2-AP) was a kind gift of Dr. Alain Chédotal.

### In ovo electroporation

In ovo electroporation was carried out according to Funahashi et al., [30] with minor adjustments. HH stage 10–11 embryos were visualised with a drop of Fast Green (0.05% in sterile PBS, Sigma). Plasmids were injected at a concentration of 1 µg/µl and were routinely co-electroporated with pCA-β-EGFPm5-U6 (Bron et al., 2004) at a concentration of 0.4 µg/µl. Following injection into the lumen of the neural tube at the level of the myelencephalon, embryos were electroporated with four 50 ms pulses of 21 V, at an interval of 100 ms. Parallel electrodes were configured with a space of 4.5 mm. Specimens were dissected, 24 hours post electroporation and fixed in 4% PFA for two hours.

### Immunohistochemistry and immunocytochemistry

Embryonic tissue was fixed for 2 hours and cells for 15 minutes in 4% PFA at 4°C. 50 µm free floating sections were collected using a vibrating microtome (Leica, model VT1000S). For collection of 20 µm cryostat sections (Bright), embryos were embedded in 1.5% Agarose/5% sucrose (Sigma), equilibrated in 30% sucrose solution overnight and frozen gradually on a metal tray cooled by contact with dry ice. Sections were mounted in ProLong Gold antifade reagent with DAPI (4′,6-diamidino 2-phenylindole) (Invitrogen). Whole embryos were washed in TBST (0.025 M Tris pH 7.5, 0.15 M NaCl, 2 mM KCl, 0.1% Tween 20), blocked overnight in 10% normal goat serum in TBST at 4°C and incubated in primary antibody in blocking solution for 6 days. Following washes in TBST and incubation in secondary antibody overnight at 4°C, whole embryos were processed for OPT. Primary antibodies used were mouse anti-Myc 9E10 (DSHB; 1∶200 dilution), mouse anti-BrdU (DSHB, 1∶100 dilution; used on chick tissue), anti-BrdU antibody (Amersham RPN202, 1∶100 dilution; used on mouse tissue), rabbit anti-GFP (Invitrogen; 1∶1000 dilution), mouse anti-Nestin (DSHB; 1∶50) and rabbit anti-Sox2 (Sigma; 1∶100). Secondary antibodies were donkey anti-rabbit Alexa 488 (Invitrogen; 1∶500 dilution) and goat anti-mouse Cy3 (Jackson Laboratories; 1∶500 dilution).

### Alkaline Phosphatase (AP) detection

AP detection on 100 µm chick sections was carried out according to Miller et al., [9].

### Cell culture

HEK293T cells were grown in 15 cm dishes in DMEM high glucose growth medium (10% FBS, 25 mM HEPES, 1x glutamine, 1x penicillin/streptomycin) and transfected with APtag5 and msPlxdc2-AP using polyethylenimine (PEI). HEK293T cells were transfected at 40% confluency and conditioned media collected for four days post transfection and concentrated using an Amicon Ultra Centrifugal Filter (50 kDa). AP activity was calculated according to Flanagan et al., [31] and AP protein concentration normalised to 300 nM with RPMI medium without serum. AP protein integrity was confirmed by western blot. Embryonic neuroepithelial cells (ENCs) were dissociated from the neural tube of E9.5 mice (MHB to rhombomere 6) according to Nardelli et al., [32]. Cells reached confluency in 7–14 days in 24-well plates and were split 1∶4. Cells used for experimental cultures were seeded on Poly-L-lysine coated coverslips, no older than passage six. In situ binding of AP proteins to cultured progenitor cells was investigated according to Flanagan et al., [31].

### Bromodeoxyuridine (BrdU) incorporation experiments in vivo and in culture

In the chick, BrdU (Sigma, 10 mM in sterile PBS) was injected into the yolk vein 24 hours after electroporation. Embryos were dissected thirty minutes after BrdU injection, fixed and processed for immunohistochemistry. BrdU-positive cell counts were carried out on a total of sixteen sections from three experimental embryos (8 sections from one specimen and 4 each from two additional specimens). Each side of the neural tube was imaged using an Apotome (Zeiss) and z-stack images were compiled. Cell counts were carried out using compressed Z stack images and ImageJ software.

In the embryonic mouse, an intraperitoneal injection of BrdU (5 mg/ml in sterile PBS) was given to pregnant females at E9.5. Embryos were dissected 1 hour following injection, fixed and processed for immunohistochemistry.

For ENC experiments, AP-conditioned media was added to cultures to a final concentration of 10 nM, 2.5 hours after splitting. BrdU (10 µg/ml) was added to the cultures 1.5 hours prior to fixing. Cells for treatment with msPlxdc2-AP and AP alone were grown from the same starter culture. Each experiment was carried out on cells cultured from at least two individual explants and was repeated three times. Duplicate coverslips per condition were used. Cell fields were photographed at five consistent locations on each coverslip. The total number of cells (DAPI) and BrdU-positive cells was calculated per condition (the sum of 10 views on 2 coverslips). The proportion of BrdU positive cells (normalised to total cell number) were compared between control and experimental conditions using Chi-squared analysis.

## Results

### Generation and analysis of *Plxdc2* mutant mice

In order to assess the functions of Plxdc2 in neural development we generated *Plxdc2* mutant animals by knocking green fluorescent protein (GFP) into the locus, replacing exon 1 and thus deleting the start codon and leader peptide (Fig. S1). Relative levels of the remaining part of the *Plxdc2* transcript (exon 7) in adult Plxdc2-GFP mice were examined by real-time PCR, which illustrated a ten-fold decrease in the cerebella of Plxdc2-GFP homozygous mutants, when compared to wildtype animals (Fig. S2) indicating that in addition to interfering with protein translation and secretion, the alteration leads to greatly reduced transcript levels from the locus. The resultant mice were homozygous viable and showed no obvious morphological or behavioural abnormalities. GFP expression in the Plxdc2-GFP mouse mirrored that previously documented in the KST37 PLAP secretory trap line [8], [9] in all areas of the E15.5 brain (Fig. S3). Detailed histological analyses of the developing and mature nervous system of homozygous mutants did not reveal defects in overall brain size, patterning, cytoarchitecture or projections of major fibre tracts (Fig. S4 and data not shown).

The functions of Plxdc2 were thus not revealed through a knock-out approach under normal conditions, at this level of analysis. The lack of detectable phenotype in *Plxdc2* mutant mice likely reflects robustness of the system rather than direct biochemical redundancy as the paralogous gene *Plxdc1* is not expressed in the developing nervous system at this stage [9]. A *Plxdc1* knockout mouse line has also been analysed and showed no obvious phenotype in the central nervous system (data not shown). Developmental systems are commonly robust to the removal of individual components [33] without necessarily implying that the protein does not have an important developmental function. In such cases, gain of function approaches can be more disruptive and informative as to the normal roles of the proteins under investigation. We therefore adopted an ectopic expression approach using in ovo electroporation to investigate the possible functions of Plxdc2.

### Plxdc2 expression in the developing head is similar in the chick and mouse

**Figure 1:**
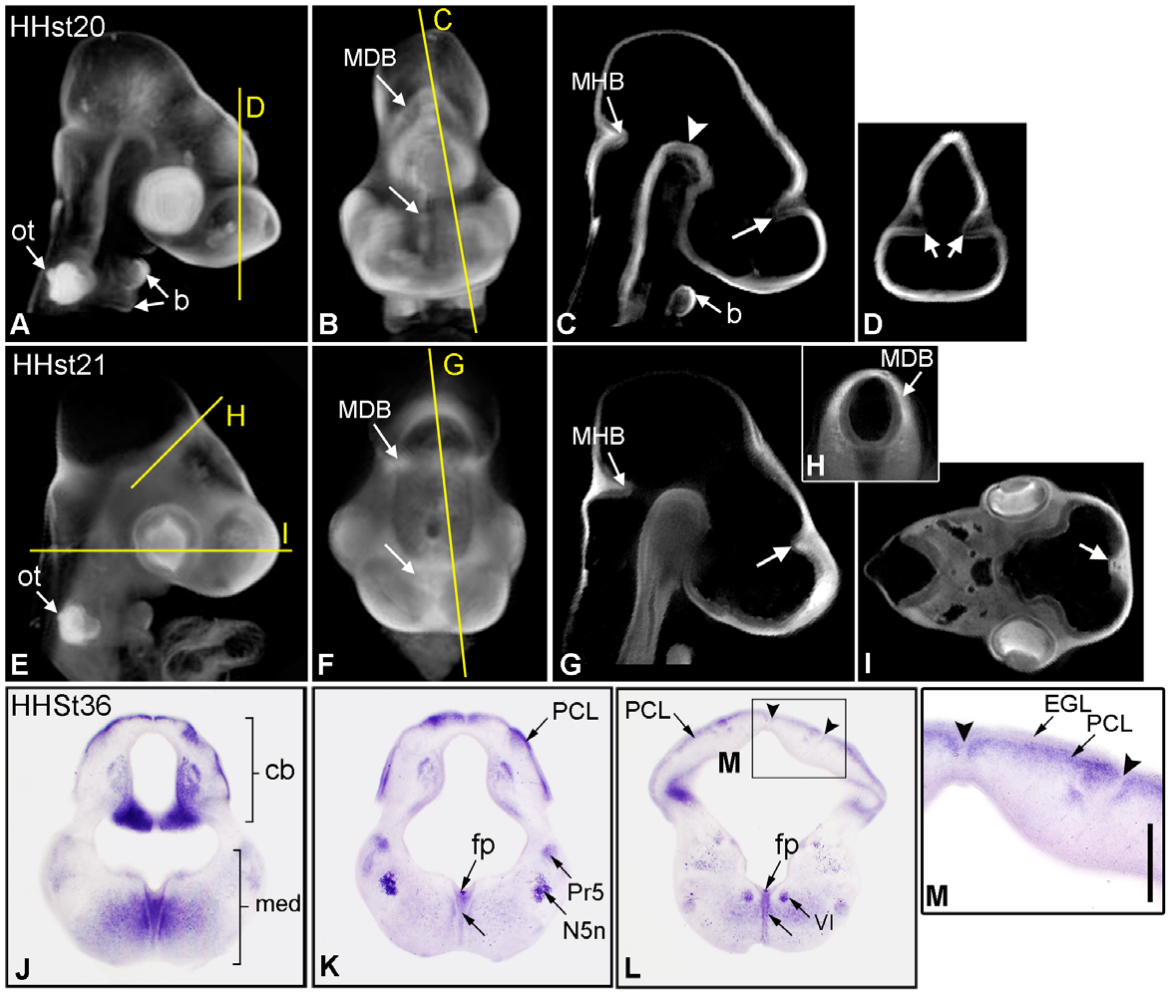
Analysis of Plxdc2 in the developing chick brain. In the chick, Plxdc2 expression was found to be similar to that previously documented in the mouse [9]. a, b, e and f, still images from different external viewing angles of volume rendered specimens showing the orientation of virtual sections in c, d and g–i. c and d, Plxdc2 expression was absent from the region of the cortical hem at HH stage 20 (arrow: b, c and d). g–i, Plxdc2 expression in the cortical hem and surrounding mesoderm was evident by HH stage 21 (arrow: f, g and i). j–l, Plxdc2 in situ hybridisation of coronal sections through the brain of a HH stage 36 chick embryo. m, higher magnification image of indicated region of l showing Plxdc2 expression in the Purkinje cell layer and a lack of Plxdc2 expression in granule cell raphes (arrowheads). Arrowhead in c, floor of midbrain; b, branchial arch expression; ot, otic vesicle; MDB, surrounding mesenchyme of the mesencephalon-diencephalon boundary; MHB, midbrain-hindbrain boundary; Arrow in k and l, Plxdc2 expression in the neuroepithelium; cb, cerebellum; fp, floor plate; med, medulla oblongata; N5n, trigeminal motor nucleus; PCL, Purkinje Cell Layer; Pr5, principle sensory trigeminal nucleus; VI, abducens nucleus.. Scale bar: a–d, 0.4 mm; e–i, 0.5 mm; j–l, 1 mm; m, 250 µm.

To assess the chick as a model for the investigation of Plxdc2 function we first confirmed similar expression to that previously demonstrated in the mouse during nervous system development [9]. *Plxdc2* expression in the chick was examined by in situ hybridisation from HH stage 16 to 21, and embryos were imaged and analysed in 3D using optical projection tomography (OPT). In several regions of the developing head, *Plxdc2* expression was found to be similar to that previously documented in the mouse, including expression at the midbrain-hindbrain boundary (MHB), the floor of the midbrain, the otic vesicles and branchial arches (Fig. 1, HH stages 20 and 21 shown).

*Plxdc2* expression was previously documented in the cortical hem of the mouse at E11.5 [9]; in the chick cortical hem *Plxdc2* expression was more complex in nature. Although the three-layer structure of the chick cortex is markedly different to the six-layered mammalian cortex [34], the major telencephalic signaling centers identified in mouse and chick are strikingly alike, suggesting a consistency between the two species in the basic patterning of the telencephalon [35]. Transient *Plxdc2* expression was first evident in the dorsal midline of the telecephalon at HH stage 17 (data not shown). At HH stage 20, *Plxdc2* expression in the forebrain of the chick was extensive throughout the dorsal diencephalon and telencephalon but absent from the region of the cortical hem (Fig. 1, c and d), with expression appearing complementary in pattern to that observed in the mouse at a comparable stage [9]. By HH stage 21, widespread *Plxdc2* expression was evident throughout the telencephalon, with intense expression at the region of the cortical hem and in the surrounding mesoderm (Fig. 1, g and i). *Plxdc2* expression was still present at the MHB and the floor of the midbrain in the HH stage 21 brain, although it was more discrete in nature than that evident at earlier stages (Fig. 1).

Although the forebrain regions of the mouse and chick are not directly comparable, the cerebellar structure and hindbrain are highly conserved between species. In the HH stage 36 chick, *Plxdc2* expression was evident in the Purkinje cell layer of the cerebellum and in several distinct nuclei within the medulla oblongata including the trigeminal motor nucleus (N5n) and the principle sensory trigeminal nucleus (Pr5) (Fig. 1, k and l). Expression in these regions, and at the neuroepithelium, corresponds to the pattern previously noted in the mouse [9].

### Plxdc2-induced thickening of the neural tube

**Figure 2:**
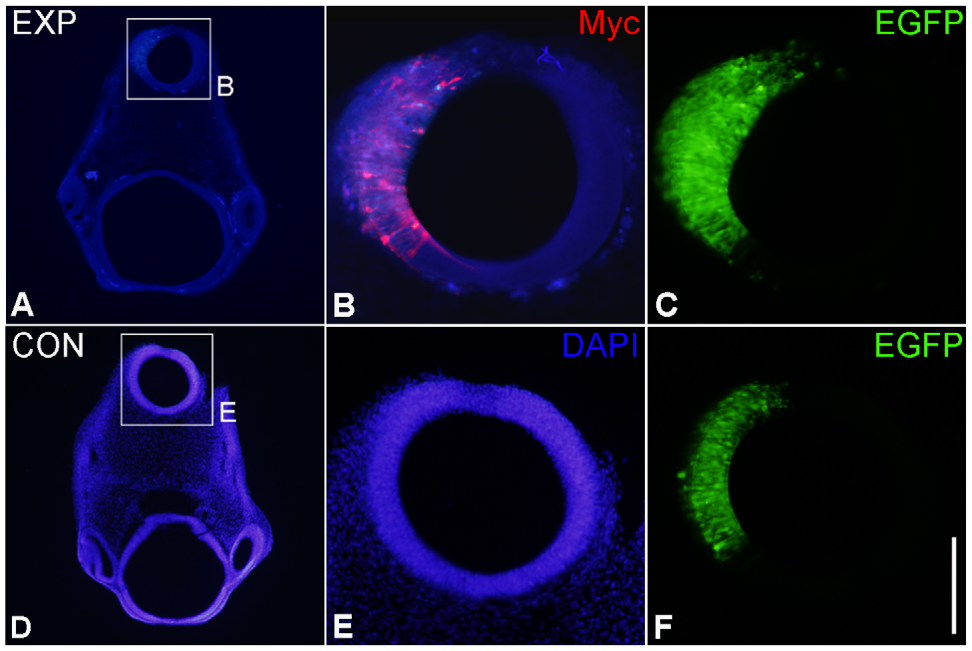
Electroporation of Plxdc2 into the HH stage 10–11 chick brain results in thickening of the neural tube on the experimental side. a–c, 50 µm transverse sections through the head of a HH stage 17 embryo, electroporated with chPlxdc2-Myc 24 hours previously. d–f, 50 µm horizontal sections through a control embryo, electroporated with pcDNA3.1myc-His(B). Empty pCA-β-EGFPm5-U6 plasmid was routinely co-electroporated (e and f). Scale bar: a and d, 400 µm; b,c,e and f, 100 µm.

**Figure 3:**
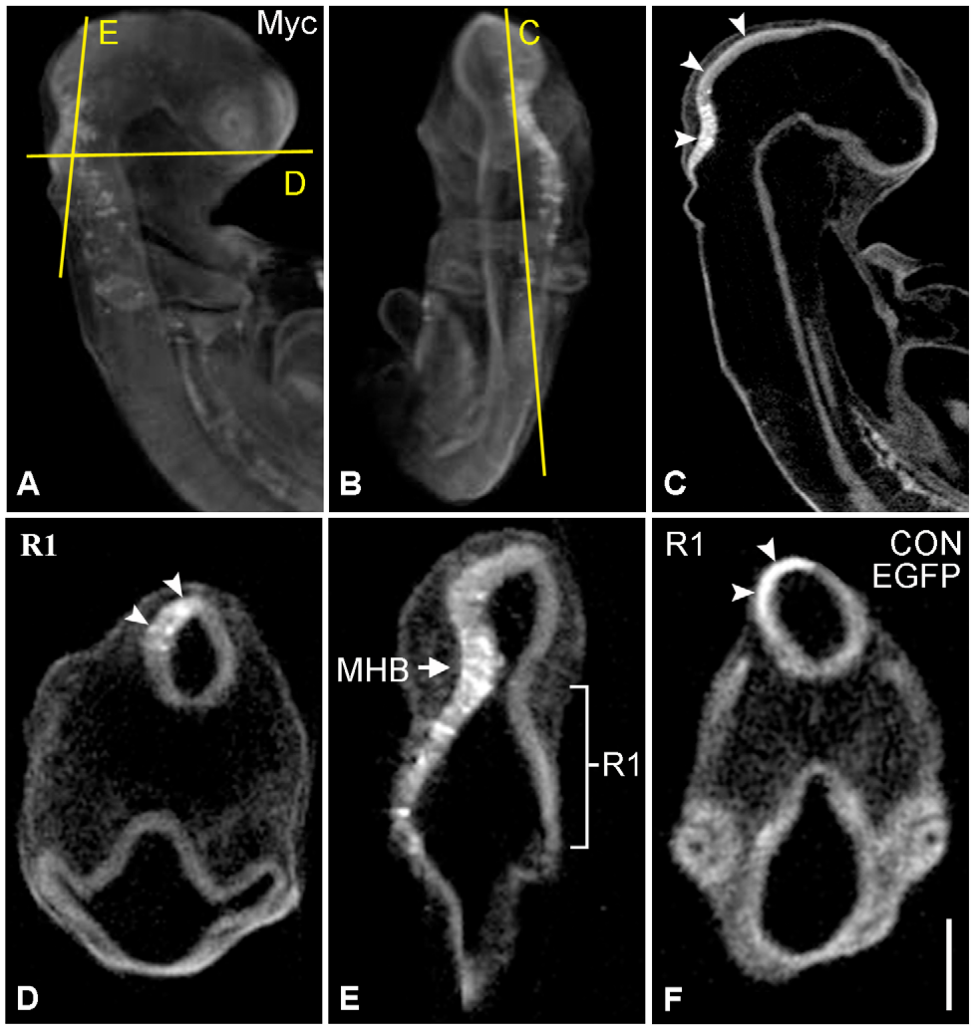
OPT analysis of a HH16 embryo electroporated with chPlxdc2-Myc 24 hours earlier. ChPlxdc2-Myc was detected by wholemount immunohistochemistry using a myc antibody (DSHB). a and b, still images from different external viewing angles of a volume rendered reconstruction (Supplemental data, movie). c–e, virtual sections through the specimen highlighting thickening of the neural tube on the experimental side (arrowheads). Planes of sectioning are indicated in a and b. f, virtual section through rhombomere 1 of a control specimen electroporated with empty pcDNA3.1myc-His(B) plasmid and pCA-β-EGFPm5-U6 under the same conditions. Thickening of the neural tube is not evident in the electroporated region (arrowheads). In control specimens, wholemount immunohistochemistry was carried out using an EGFP antibody (Invitrogen). MHB, midbrain-hindbrain boundary; R1, rhombomere 1. Scale bar: a–c, 0.4 mm; d and e, 0.25 mm; f, 0.2 mm.

An investigation of the effects of misexpression of Plxdc2 in the chick brain was carried out by means of in ovo electroporation of a full length chick Plxdc2 expression construct, chPlxdc2-Myc. Initial analysis of sections through the embryo, visualising the Myc tag and co-electroporated EGFP by immunohistochemical techniques, revealed that expression of chPlxdc2-Myc in the neural tube resulted in apparent thickening on the experimental side (Fig. 2b), compared to controls. To confirm and quantify the thickening effect in the chick, 3D OPT imaging was employed, following wholemount immunohistochemistry with antibodies against Myc and EGFP. The advantage of the 3D digital OPT data was to allow precise orientation of the planes of section through the developing brain [36]. Thickening of the neural tube was throughout the region of chPlxdc2-Myc expression (Fig. 3, c–e).

**Figure 4:**
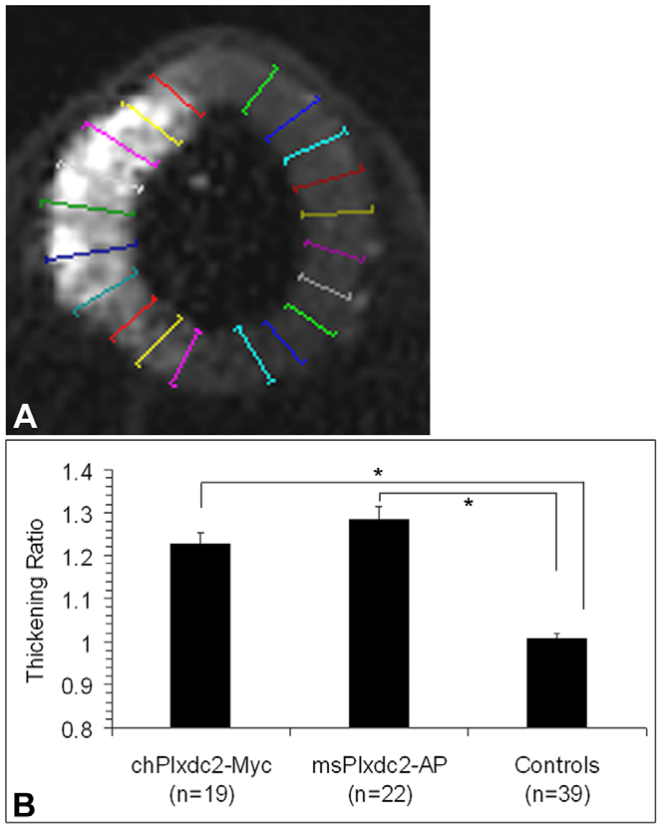
Statistical analysis of the Plxdc2-induced neural tube thickening phenotype. a, Representation of the ten measurements taken through each side of the neural tube. b, Plot of the mean thickening ratios across in ovo electroporation experiments. Controls across experimental parameters were found to provide consistent thickening ratios and were pooled. n =  number of sections analysed.

Comprehensive analysis of the region of chPlxdc2-Myc expression and corresponding neural tube thickening was carried out using OPT. In a study of seven specimens electroporated with chPlxdc2-Myc and analysed by OPT, all were found to exhibit significant neural tube thickening. Electroporation affects the rate of normal development in experimental animals and, 24 hours post electroporation, embryos ranged from HH stage 15 to HH stage 20. Therefore, absolute measurements of neural tube width could not be compared across specimens. To circumvent this problem, average thickness of the experimental side of the neural tube was divided by average thickness of the control side, resulting in a standardised ‘thickening ratio’. Means were based on ten measurements taken through either side of the neural tube (Fig. 4a). Statistical analysis of thickening ratios highlighted a significant increase in neural tube thickness following misexpression of chPlxdc2 (Fig. 4b; 19 sections taken from 7 specimens) when compared to control embryos (39 sections from 10 specimens; Independent t-test, p≤0.0001).

**Figure 5:**
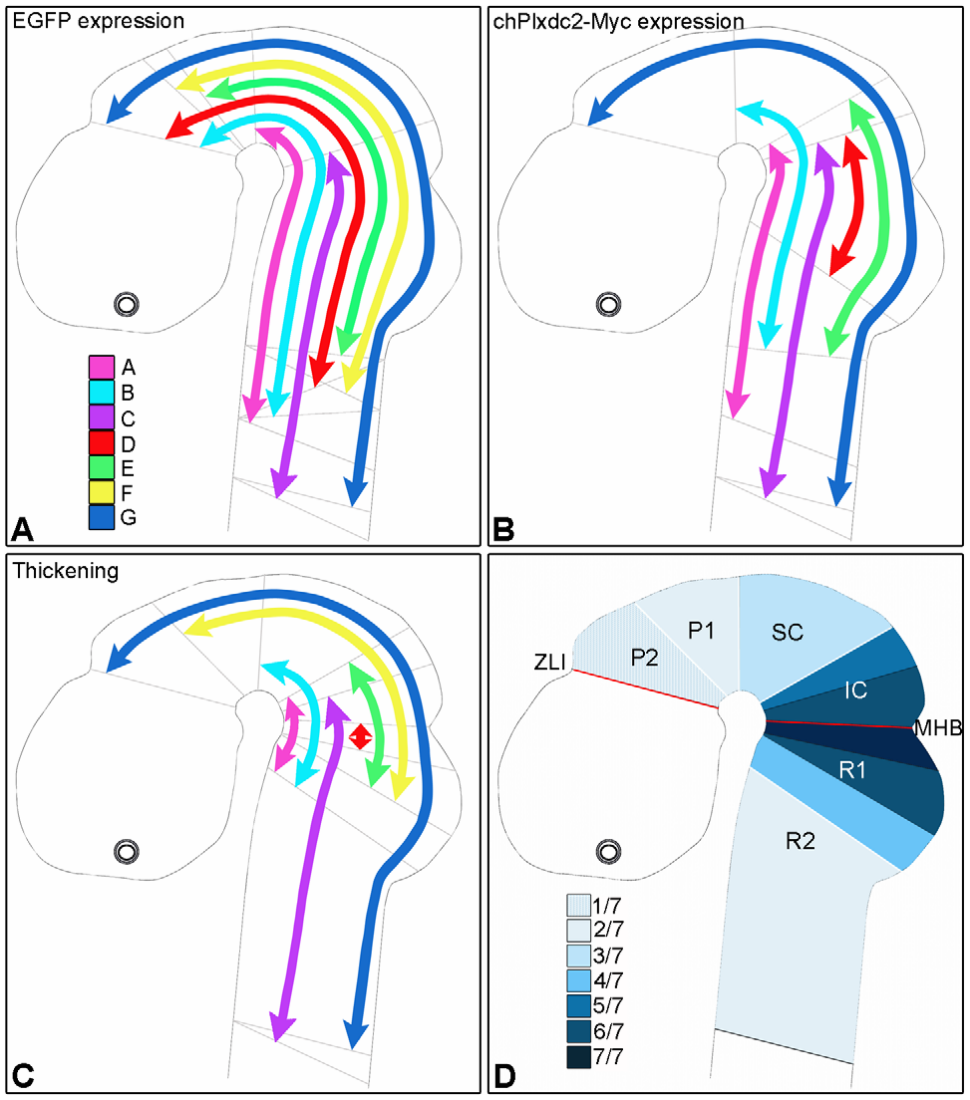
Summary of results from OPT analysis of chPlxdc2-Myc expression and neural tube thickening in seven embryos (A–G), 24 hours post electroporation. a, Extent of EGFP expressing cells; b, corresponding extent of chPlxdc2-Myc expression (chPlxdc2-Myc data was not obtained for specimen F); c, extent of neural tube thickening in each of seven specimens; d, occurence of thickening in individual brain regions. Grey lines in a-c represent the limits of expression/thickening. IC, inferior colliculus; MHB, midbrain-hindbrain boundary; P1, prosomere 1; P2, prosomere 2; R1, rhombomere 1; R2, rhombomere 2; SC, superior colliculus; ZLI, Zona Limitans Intrathalamica.

The spatial domains showing expression of EGFP, chPlxdc2-Myc and corresponding thickening in these embryos were mapped to a representation of the chick neural tube at this stage of development (Fig. 5). In general, the region of detectable chPlxdc2-Myc expression was more restricted than that of EGFP with the exceptions of specimens C and G (Fig. 5, a and b). Differences in the extent of EGFP and chPlxdc2-Myc expression could be due to differences in the efficiency of marker detection. In locations where both markers were detected there was correspondence between cells taking up and expressing the two constructs. Plxdc2-induced neural tube thickening was not confined to any one region of the developing brain, but was correlated with expression of the chPlxdc2-Myc plasmid (Fig. 5, b and c). Thickening of the neural tube was evident in regions spanning from the zona limitans intrathalamica (ZLI) to rhombomere 2 and more posteriorly into the spinal cord. However, neural tube thickening was observed most commonly in the region surrounding the MHB and decreased gradually in an anterior or posterior direction (Fig. 5d). This could be due to the placement of electrodes during in ovo electroporation. However, of five specimens exhibiting chPlxdc2-Myc expression in the region of rhombomere 2 and more posteriorly, only two exhibited clear thickening in this area (Fig. 5, b and c). This could be indicative of a regional limitation of the phenotype due to differential expression of interacting proteins.

### Extracellular Plxdc2 can induce neural tube thickening

**Figure 6:**
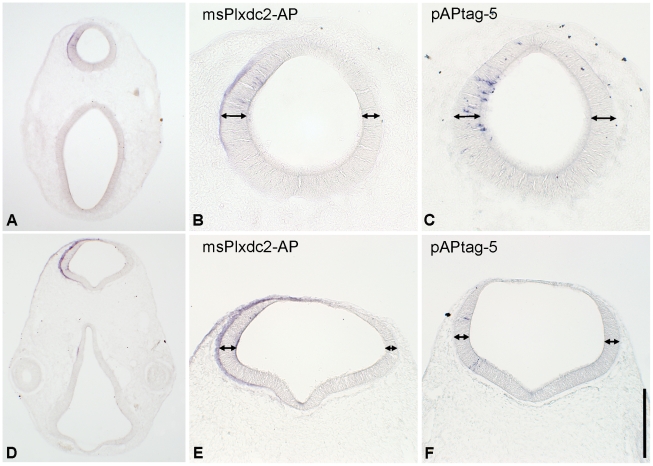
Expression of Plxdc2-AP(CT) in the chick brain causes thickening of the neural tube on the experimental side. a and d, 20 µm cryostat sections through chick embryos electroporated with Plxdc2-AP, 24 hours previously. b and e, higher magnification images highlighting neural tube thickening on the experimental side. c and f, 30 µm cryostat sections through control specimens electroporated with the empty pAPtag5 plasmid. Scale bar: a and d, 400 µm; b and c, 100 µm; e and f, 200 µm.

Shedding of the ectodomain of Plxdc2 (which includes the region of nidogen homology and the PSI domain) is mediated by a metalloprotease, while the remaining section of the protein is a target for cleavage by γ-secretase [10], [11]. To determine whether Plxdc2 acts in a cell non-autonomous manner through its ectodomain, msPlxdc2-AP, which encodes the extracellular portion of the mouse Plxdc2 protein, tagged with AP at the C-terminal, was electroporated into the chick neural tube (Fig. 6 a, b, d and e). Colorimetric detection of the AP tag highlighted a similar phenotype to that observed in chPlxdc2-Myc expressing embryos, a significant increase in neural tube thickness following misexpression of msPlxdc2-AP (Fig. 4b; 22 sections taken from 5 specimens) when compared to control embryos (39 sections from 10 specimens; Independent T-test, p≤0.0001). This result indicates that Plxdc2 functions in a cell non-autonomous manner, most likely acting as a ligand and that the biochemical function of the protein is conserved between species.

### Plxdc2 misexpression increases proliferation and alters neurogenesis

**Figure 7:**
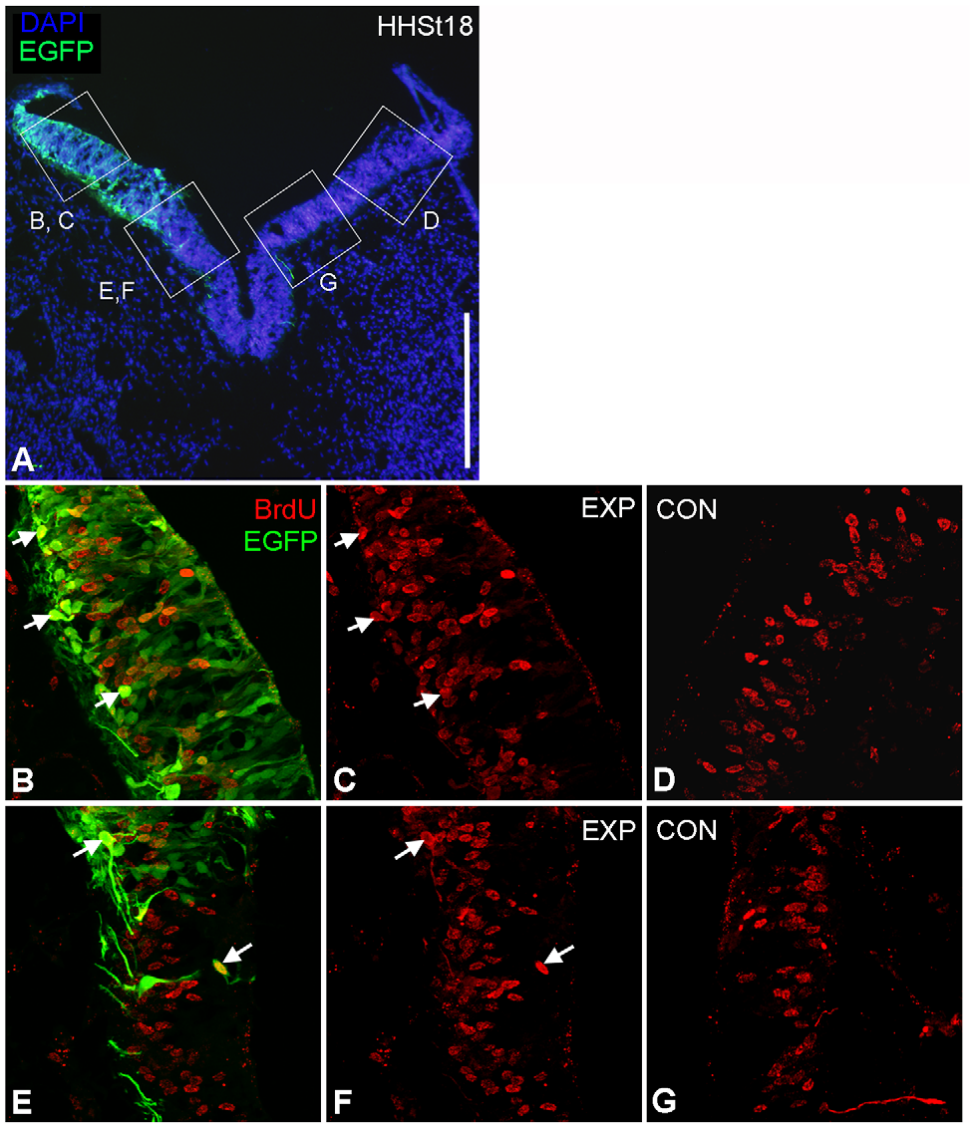
Examination of BrdU-positive cells in the neural tube of a HH stage 18 chick ectopically expressing chPlxdc2-myc illustrates an increased number of BrdU positive cells on the electroporated side. a, low magnification image of a transverse section of the neural tube in the region of rhombomere 1/2 highlighting the regions shown in b–g. b–g, compressed confocal z-stack images through the neural tube at higher magnification. b and e, overlay of EGFP expression and BrdU incorporation on the experimental side of the neural tube showing examples of cells co-expressing EGFP and BrdU (arrows). Apparent ectopic proliferation in the VZ evident in this section was not evident in other sections (this specimen was chosen as the clearest example of increased BrdU incorporation). Ectopic proliferating cells in the c, d, f and g, BrdU incorporation on either side of the neural tube. Scale bar: a, 200 µm; b–g, 50 µm.

To investigate the cause of the increase in neural tube width, markers of cell proliferation, cell death and neurogenesis were examined at the site of electroporation with chPlxdc2-Myc. BrdU incorporation highlighted an increase in the number of BrdU-positive cells on the experimental side (Fig. 7, c to g). EGFP expression from the co-electroporated reporter plasmid was used to highlight electroporated cells. In some instances EGFP-expressing cells were positive for BrdU, although this was not exclusively the case. Many EGFP-positive cells did not incorporate BrdU (Fig. 7, b and e). Statistical analysis of BrdU-positive cell counts confirmed a significant increase in BrdU-positive cells on the chPlxdc2-Myc-expressing side of the neural tube (paired T-Test, p≤0.0001; average increase of 49.3 BrdU-positive cells on the experimental side). No correlation in thickening ratio and increase in number of BrdU-positive cells was observed (data not shown). Cell size was investigated 24 hours following electroporation and comparison with controls illustrated no increase following Plxdc2 misexpression (data not shown).

The effect of Plxdc2 misexpression on cell death in the neural tube was also investigated. There was no significant difference in the number of Caspase3-positive cells on the experimental versus the control side of the neural tube 24 hours following Plxdc2 misexpression (data not shown).

**Figure 8:**
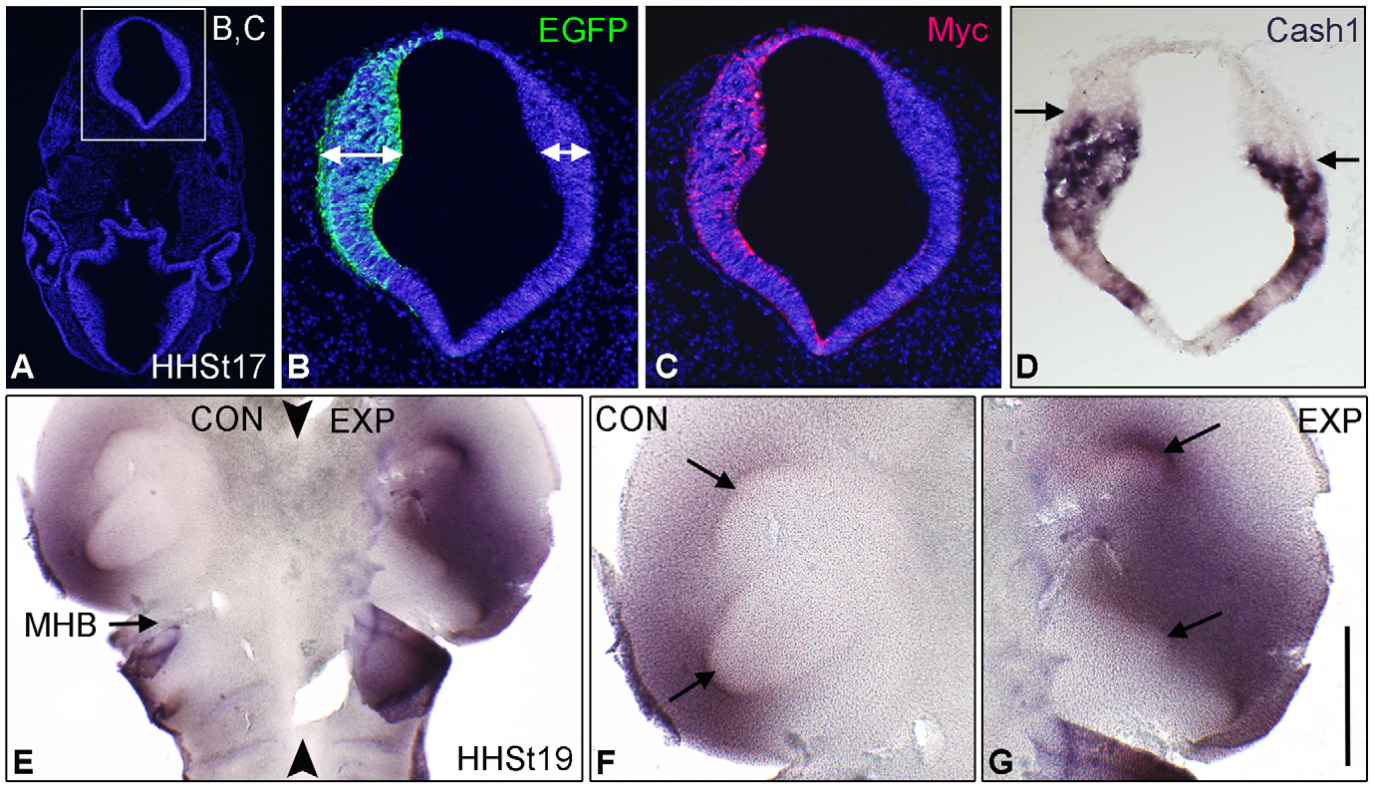
Plxdc2 misexpression in the neural tube of the chick results in disruption of the normal expression pattern of Cash1. a–c, immunohistochemistry of coronal sections illustrating Plxdc2-Myc and EGFP expression (double headed arrows highlight neural tube thickening on the experimental side). d, adjacent coronal section through the same embryo following Cash1 in situ hybridisation (Arrows highlight the displacement of Cash1 expression on the experimental side). e, ‘open book’ preparation of the neural tube following wholemount Cash1 in situ hybridisation (Arrowheads, ventral midline). Arrows in f, mesencephalic regions lacking Cash1 expression. Arrows in g, displacement of regions marked in f by expression of chPlxdc2-Myc. Scale bar: b, c, and d 115 µm; e, 300 µm; f and g, 200 µm.

The effect of chPlxdc2-Myc expression on neurogenesis within the neural tube was investigated by examining the expression of the proneural gene Cash1. In a study of thirteen embryos, Plxdc2 misexpression in the posterior diencephalon and mesencephalon of eight specimens caused a visible increase in Cash1 expression levels when compared to the control side. In three instances, misexpression of Plxdc2 caused a dorsal-ventral expansion in the normal expression pattern of Cash1, resulting in expression of the gene in regions of the neural tube which normally lack Cash1 expression (Fig. 8, d to g). These results indicate an increase in the number of differentiating neurons at this stage. In the remaining 2 embryos, and in control embryos (electroporated with empty pcDNA3.1myc-His(B) plasmid and EGFP) no difference in Cash1 expression was observed.

To examine the effect chPlxdc2-Myc misexpression has on the brain at later stages of development, embryos were dissected five and ten days following electroporation (E7 and E12 respectively). External examination of experimental specimens (E7) and dissected brains (E12) illustrated no gross morphological effect five or ten days following electroporation (Fig. S5). Both sides of the brain appeared to be of appropriate size with no unusual features noted at a gross level or by cresyl violet staining. The lack of morphological defects in the midbrains of older embryos suggests that early increases in proliferation and Cash1 expression do not have a permanent effect on progenitor or neuronal cell number.

### Plxdc2 knockdown does not result in abnormal proliferation

Following the discovery of Plxdc2-induced effects on proliferation and neurogenesis in the developing neural tube of the chick, we investigated the effect of knockdown of endogenous Plxdc2 in the chick and mouse. Knockdown of endogenous Plxdc2 in the chick was investigated by electroporation of shRNAs into the neural tube of HH stage 10–11 embryos. No effect on neural tube thickness at the MHB, or in any other region, was observed following electroporation of Plxdc2 specific shRNAs (Fig. S6). However, co-electroporation of shRNAs and chPlxdc2-Myc did result in the rescue of the neural tube thickening phenotype observed when chPlxdc2-Myc was electroporated alone (Fig. S6).

**Figure 9:**
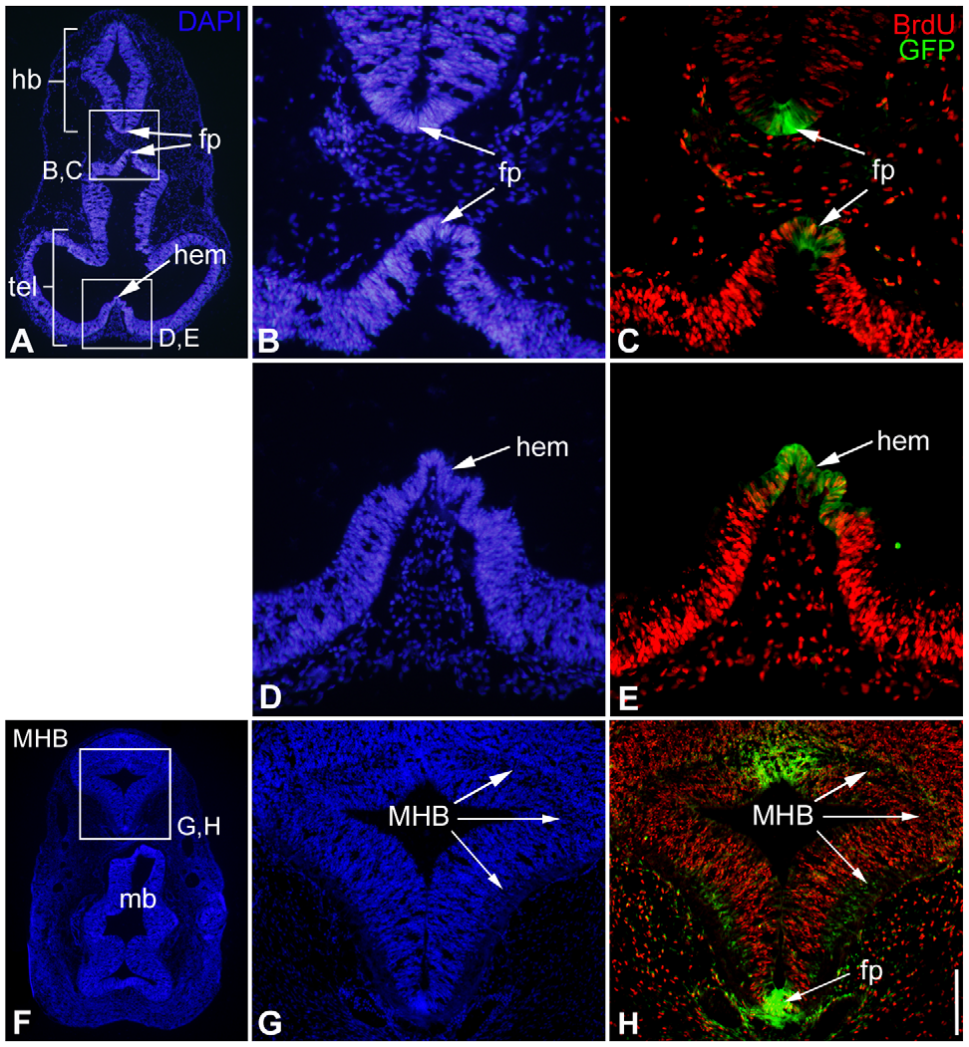
Expression of Plxdc2 in regions of restricted proliferation. a–h, horizontal sections through the head of an E10.5 Plxdc2GFP heterozygous mutant showing Plxdc2GFP expression (c, e and h; in green) in the floorplate (fp), cortical hem and MHB. Fewer BrdU-positive cells are present in regions of Plxdc2 expression relative to surrounding regions. hb, hindbrain; mb, midbrain; tel, telencephalon. Scale bar: a, 500 µm; b, c, d and e, 100 µm; g and h, 200 µm; f, 750 µm.

**Figure 10:**
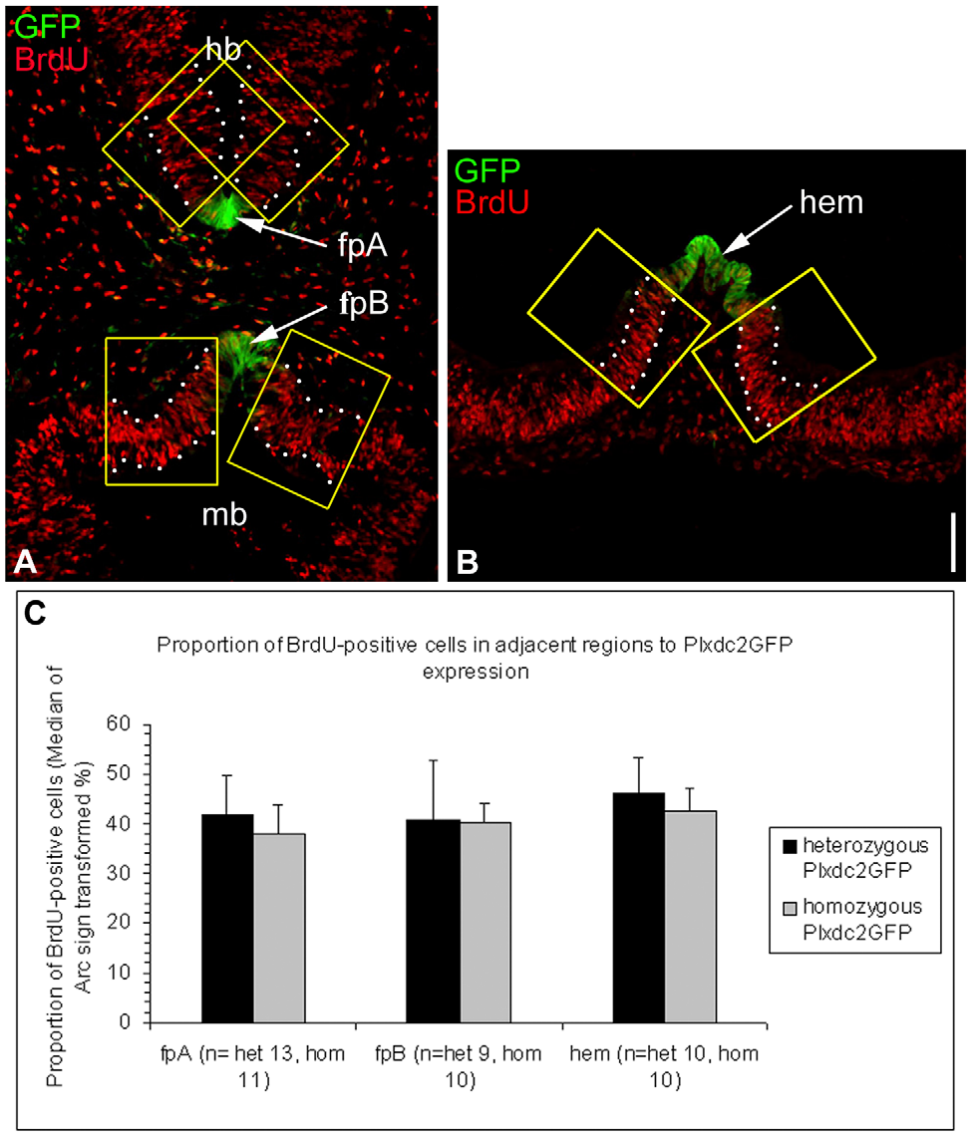
Homozygous Plxdc2GFP mutants do not exhibit reduced rates of proliferation in regions of the neural tube surrounding the floorplate and cortical hem. a and b, representative images of horizontal sections through a heterozygous Plxdc2GFP mouse illustrating areas of analysis of the proportion of BrdU-positive cells within the dotted borders on both sides of the region of normal Plxdc2 expression in the floorplate (fp) and cortical hem. (a) In order to carry out regional investigation, the floorplate was divided into two regions, fpA and fpB. c) Statistical analysis illustrated no difference in the proportion of BrdU positive cells in homozygous Plxdc2GFP mice in regions of the neural tube surrounding the floorplate and cortical hem. Data were Arc Sign Transformed and a Mann Whitney U-test carried out due to the non-parametric nature of BrdU data sets. Scale Bar: 100 µm.

In the mouse, proliferation was examined in the neural tube of homozygous Plxdc2GFP mice at E10.5 by analysis of BrdU incorporation. In many instances Plxdc2 is expressed in the brain in regions of reduced proliferation. These regions include major patterning centres of the early brain such as the floorplate, the cortical hem and the MHB (Fig. 9). Expression of secreted mitogens from regions of restricted proliferation has been well documented [6], [9], [35], [37], [38], [39], [40]. Analysis of BrdU-positive cells in Plxdc2GFP mice was carried out in regions of the neural tube adjacent to regions of Plxdc2 expression in the floorplate and cortical hem. The number of BrdU-positive cells in the neural tube was counted in a set area on both sides of Plxdc2GFP expression and normalised to total cell number as marked by DAPI staining for both the floor plate (Fig. 10a) and the cortical hem (Fig. 10b). There was no significant difference in the proportion of BrdU-positive cells in homozygous animals when compared to heterozygous controls in regions surrounding the cortical hem and floorplate (Wilcoxon Rank Sum Test; p = 0.840 (fpA), p = 0.327 (fpB), p = 0.983 (hem)).

### Plxdc2 has a mitogenic effect on ENCs in culture

**Figure 11:**
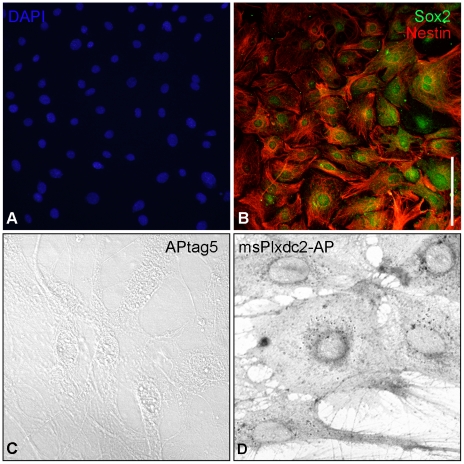
Characterisation of ENCs cultured from the mouse E9.5 neural tube. In accordance with previously published data, ENCs cultured from the mouse neural tube were shown to express Nestin and Sox2 (a & b). AP binding assays demonstrated that msPlxdc2-AP (d) but not AP alone (c) bound to ENCs. Scale bar: a and b, 200 µm; c and d 100 µm.

To test more directly for conservation of the proliferative activity of Plxdc2 in the mouse, an in vitro culture system was adopted. Embryonic neuroepithelial cells (ENCs) were cultured from the neural tube of E9.5 mice (from the region of the MHB to rhombomere 6) and were characterized with antibodies to several progenitor and neural cell markers [32]. In agreement with published data, the cells expressed Nestin and Sox2 (Fig. 11, a and b; [41]), but did not express the proneural marker, Mash1 or the post-mitotic neural marker TUJI (data not shown). ENCs of this kind have been shown to have a low rate of proliferation in normal conditions and can take 7–21 days to reach confluence in a 24-well dish [32], [41]. Our cultures illustrated similar behaviour. Initial survival of cultures was 76% 24 hours after explantation. However, cells from very few explants survived longer than 6 passages in culture, and those that did exhibited altered rates of growth and behaviour. For this reason, all experiments were carried out on cultures prior to passage 6.

**Figure 12:**
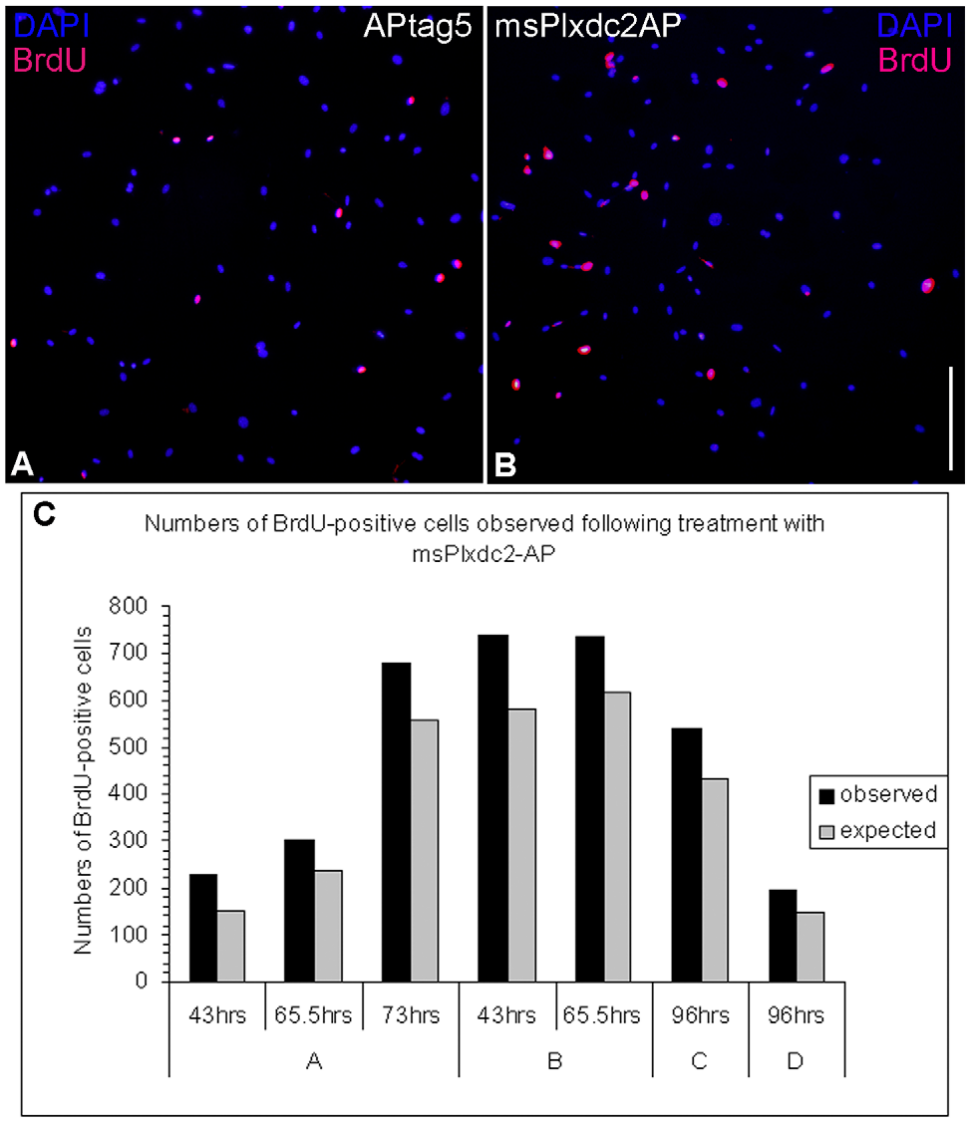
Treatment with extracellular msPlxdc2-AP results in an increase in proliferation in ENCs cultured from the E9.5 neural tube. Immunocytochemistry with an antibody to BrdU illustrated an increase in the number of BrdU-positive cells following treatment with msPlxdc2-AP (a and b). c) Total number of BrdU-positive cells observed in msPlxdc2-AP cultures originating from four individual neural tubes (A–D). Observed numbers are shown alongside expected values calculated from the proportion of BrdU-positive cells in APtag5 treated controls. Times stated refer to the period of treatment with conditioned media. Chi-squared analyses showed a significant increase in the proportion of BrdU-positive cells in msPlxdc2-AP treated cultures (Chi square results: A, χ^2^ = 41.37 (43 hrs), 20.52 (65.5 hrs) and 27.73 (73 hrs); B, χ^2^ = 44.31(43 hrs) and 24.49(65.5 hrs); Cχ^2^ = 29.03; D, χ^2^ = 16.39; df = 1, p<0.001 in all instances). Scale bar: 300 µm.

AP-binding assays indicated that msPlxdc2-AP but not AP alone bound to ENCs (Fig. 11, c and d). To investigate the effect of Plxdc2 on proliferation of ENCs, cultures were treated with msPlxdc2-AP and grown for a maximum of four days (Fig. 12). There was a significant increase in the number of BrdU-positive cells following treatment with msPlxdc2-AP when compared to controls treated with AP alone. (Fig. 12, a and b; Table S1). Cells treated with msPlxdc2-AP exhibited a ∼20–50% increase in proliferation when compared to controls across all time-points analysed (Table S2).

Markers of neuronal differentiation were analysed in ENCS following treatment with msPlxdc2-AP. Sox2 and nestin expression remained constant. In addition, no Mash1 or TUJI expression was observed in ENCs post treatment (data not shown).

## Discussion

These studies demonstrate that Plxdc2 is a novel mitogen for neural progenitors in the developing neural tube with the ability to alter normal patterns of neurogenesis. Expression in a number of patterning centres in the developing brain is consistent with a role in the coordinated control of proliferation and differentiation across the neuraxis. In particular, *Plxdc2* is expressed in the cortical hem, MHB, floor plate and dorsal midline. These patterning centres are intimately involved in the coordination of proliferation and cell fate specification in the developing neural tube through the secretion of mitogens and morphogens into adjacent regions. Cleavage and shedding of the extracellular domain of Plxdc2 has been demonstrated in vitro [10], [11]. In line with this finding, we have demonstrated that expression of a secreted form of Plxdc2 results in neural tube thickening in the chick. In addition, treatment of murine ENCs with the extracellular form of the Plxdc2 protein also results in increased proliferation in vitro.

These effects are presumably mediated through binding of the extracellular domain of Plxdc2 to an unidentified receptor. At least one such receptor is clearly expressed by early murine neural tube progenitors as demonstrated by Plxdc2-AP-binding activity. Although no definitive binding partner for Plxdc2 has been identified, several studies have isolated nidogen as a binding partner for the related protein, Plxdc1 [42], [43]. As nidogen is a secreted protein of the extracellular matrix, it seems highly unlikely that it could fulfill a receptor function. However, if it binds Plxdc2 it could conceivably play some role in sequestering, stabilising or presenting the shed Plxdc2 ectodomain. In vitro studies have also identified cortactin as a putative binding protein of both Plxdc2 and Plxdc1 [44]. However, cortactin is a cytoplasmic molecule and the interaction with the extracellular domains of Plxdc1 and 2 demonstrated in this study thus seems unlikely to be physiological.

Early misexpression of Plxdc2 in the chick neural tube did not result in a persistent phenotype at later stages of development. In similar experiments in the chick, misexpression of Lmx1b and Wnt1 results in drastic expansion of the tectum, torus semicircularis and rhombic lip (Lmx1b misexpression) and in the development of addition folia in the cerebellum (Wnt1 misexpression) [45]. In both these cases, misexpression of the gene of interest repressed normal expression of Cash1 (by 48 hours post Lmx1b electroporation and 24 hours post Wnt1 electroporation [45]). Excess proliferation thus most likely resulted in an expanded pool of progenitors and subsequent overgrowth. In the case of Plxdc2, misexpression of the gene in the chick was not accompanied by reduction in expression of Cash1, but by increased Cash1 levels and a dorsal-ventral expansion of Cash1 expression 24 hours following electroporation. Cash1 expression forces cells out of a proliferative state and toward differentiation, marking a transient stage of the cell as it differentiates into a neuron [26]. Thus, excess proliferation at early stages due to Plxdc2 over-expression was accompanied by the precocious production of differentiating neurons, and a temporary thickening of the neural tube, without necessarily leading to an increase in the eventual number.

Increased levels of Cash1 could either be a direct effect of Plxdc2 or secondary to increased proliferation. Similarly, dorsal-ventral expansion of Cash1-positive zones in the neural tube could be due to the migration of additional Cash1-positive differentiating neurons. It thus remains unclear whether Plxdc2 directly affects neurogenesis per se or whether the alterations in Cash1 expression in fact simply reflect alterations in proliferation without a concomitant block on neurogenesis.

The lack of correlation between the number of BrdU-positive cells and the degree of thickening in each specimen may be due to the fact that the BrdU from one injection is present for only a fraction of the time (half an hour) over which the neural tube is exposed to Plxdc2 over-expression (24 hours). Our examination of BrdU incorporation thus provides only a snap shot of proliferation over a brief time period and not a quantitative measure of the total increase in proliferation. No changes in cell size following Plxdc2 misexpression were observed, excluding this potential mechanism as the possible cause of neural tube thickening.

In culture, treatment of ENCs with msPlxdc2-AP did not result in any change in the expression of differentiation markers such as Mash1 and TUJI. Experiments analysing the effects of Gata2 misexpression in vivo and in culture have yielded similar results [41]. In vivo, Gata2 misexpression causes decreased proliferation along with decreased Sox2 expression and increased TUJI and neurofilament expression [41]. In ENCs, however, differences in Sox2, TUJI and neurofilament are not observed following Gata2 misexpression [41]. These ENCs may thus not be fully competent to differentiate under these culture conditions. Thus, while the effects of Plxdc2 on their proliferation are clear, the lack of induction of differentiation markers must be interpreted with caution. Plxdc2 alone may not be sufficient to enable these cells to progress along the differentiation pathway, which may require further molecular events absent in our culture conditions.

In this light, it is interesting to consider the similarity in the expression pattern of *Plxdc2* with those of important secreted morphogens of the early embryo such as members of the Wnt family [9]. Members of the Wnt family have been implicated in the maintanance of progenitor pools in the developing neural tube and the control of neural differentiation [6], [45], [46], [47]. Wnt proteins do not always affect proliferation at the expense of differentiation and vice versa. In a similar manner to our findings for Plxdc2 in vivo, expression of Wnt3a and/or Wnt5a, in postnatal or adult NPCs in culture results in increased proliferation and neuronal differentiation [48]. Interestingly, Wnt3a and Wnt5a are co-expressed with Plxdc2 in the cortical hem and MHB respectively [9]. It is clear that the pathways stimulated and the effect of individual Wnts can vary depending on the cellular context [49] making the possible intersection of Plxdc2 with Wnt signaling pathways an important topic for further study.

The demonstrated effects of Plxdc2 on proliferation in the chick neural tube and in ENCs in vitro and its expression in various patterning centres suggests that endogenous Plxdc2, through shedding of its extracellular domain, could contribute to the regulation of proliferation in the neural tube in vivo. The finding that proliferation is overtly normal in *Plxdc2* knockout mice indicates, however, that the contribution of Plxdc2 to control of this process can be compensated for, at least under normal conditions. The functions of many proteins that appear to be redundant under typical conditions may be revealed under conditions where the system is sensitised or stressed. We thus hypothesise that a requirement for Plxdc2 function in vivo may be revealed in situations where the functions of interacting proteins or converging signaling pathways are perturbed.

For instance, an in vivo function of Plxdc2 may be evident in contexts where expression of the gene is altered. There is convergent evidence from several sources that the expression of Plxdc2 may be dynamically regulated in response to cellular stresses or in unusual cellular contexts, including cancer. Plxdc2 (TEM7R) is strongly expressed in endothelial cells of stroma of human colorectal tumours and mouse melanoma but not of surrounding normal tissue [12]. It has also been found by RT-PCR to be upregulated in human colorectal and breast cancer samples [12], [16], [17]. Also, CpG islands in the *Plxdc2* promoter show large methylation differences between subtypes of acute lymphoblastic leukemia and *Plxdc2* is one of 40 genes whose methylation status strongly predicts prognosis [50]. *Plxdc2* was also one of a set of genes whose expression was altered synergistically in murine colon cells expressing oncogenic mutant forms of both p53 and Ras [19]. Among these genes, *Plxdc2* showed the strongest level of reduction in response to the combined oncogenic mutations. While this would seem to contradict a role in promoting cell proliferation, downregulation of Plxdc2 could in fact be part of an antiproliferative cellular stress response known to be induced by oncogenic mutations. In support of such a role, restoring wild-type levels of Plxdc2 expression increased tumourigenicity of transplanted oncogenic p53/Ras-transfected cells. This experiment clearly demonstrates that loss of function of Plxdc2 can indeed have an effect in a sensitised context. The decreased tumorigenicity of the transformed cells attributable to reduction of Plxdc2 expression is consistent with our findings that Plxdc2 is a mitogenic factor.

Conversely, Plxdc2 is also upregulated in human prostate epithelial cells undergoing senescence, cellular arrest or apoptosis [20]. It is interesting to note that this profile is shared with Wnt5a in these cells. In addition, Plxdc2 is upregulated in rat fibroblasts by the Rb/E2F1 pathway but repressed by PI3K/Akt signaling [18]. Genes with this profile may be involved in the promotion of cell death in response to E2F1 activation, which is blocked by concomitant PI3K activation, tilting the balance towards proliferation over apoptosis. These findings suggest additional complexity and context-dependence in the functions of Plxdc2. They may also reflect distinct cell-autonomous functions of Plxdc2 rather than the cell non-autonomous effects we describe here.

Our findings identify the first cellular function for the Plxdc2 protein and implicate it as an additional component in the network of proteins regulating proliferation and differentiation in the developing nervous system. They also provide the first mechanistic insights into the possible role of this gene in various cancers and other cellular contexts where it has been previously implicated.

## Supporting Information

Figure S1 Design strategy for the creation of the Plxdc2GFP mouse line. The Plxdc2 gene is inactivated through replacement of the start codon & leader peptide by a 5S-EGFP-pA cassette following homologous recombination in ES cells.
(9.84 MB TIF)

Figure S2 Examination of residual (exon 7) Plxdc2 transcript levels in Plxdc2GFP mice by realtime PCR. Samples for real time PCR were normalised to the mouse RPO gene. Plxdc2 transcript levels were significantly reduced in heterozygous Plxdc2GFP mice when compared to wildtype animals (independent T-test, p≤0.0001). Plxdc2 expression in heterozygous mutants was approximately half that evident in wildtype mice. Plxdc2 transcript levels were significantly reduced in homozygous Plxdc2GFP mice when compared to those in heterozygous animals (independent T-test, p≤0.0001). There was approximately a ten fold decrease in Plxdc2 transcript levels in the cerebella of Plxdc2GFP homozygous mutants, when compared to wildtype animals showing that in addition to abolishing normal protein translation and secretion (start codon and leader sequence removed), transcript levels from the locus are also greatly reduced by the genomic alteration.
(1.73 MB TIF)

Figure S3 Plxdc2 expression in the E15.5 Plxdc2GFP mouse brain. GFP expression was compared to that of Plxdc2-βgeo in heterozygous PLAP secretory trap mice at the same stage of development. Plxdc2 expression in the Plxdc2GFP mouse line mirrored that in the PLAP secretory trap line in all areas of the E15.5 brain. Representative images through the brain are shown illustrating GFP expression in many regions of the E15.5 brain including the glial wedge (GW), fimbria (fim), dentate gyrus (DG), caudate putamen (CP), midbrain reticular formation (MRF), floor plate (fp), principle sensory trigeminal nucleus (Pr5), Purkinje cell layer (PCL) and vestibular nuclei (vn). a,c,e,g and i: coronal sections through the brain of a heterozygous Plxdc2 gene trap mouse illustrating Plxdc2-βgeo expression. b,d,f,h and j: corresponding coronal sections through the brain of a heterozygous Plxdc2GFP mouse illustrating GFP expression. arrow in a and b, Plxdc2 expression at the medial septum; arrowhead in g and h, clusters of Plxdc2 expression at the border region of the tectum and the pons; cb, cerebellum; cx, cortex; hip, hippocampus; hy, hypothalamus; ic, inferior colliculus; med, medulla oblongata; sc, superior colliculus; sep; septum; teg, tegmentum; th, thalamus. Scale bar:1 mm.
(5.74 MB TIF)

Figure S4 Cresyl violet staining of coronal sections through the brain of E15.5 Plxdc2GFP mice. No gross morphological phenotype was evident in Plxdc2GFP homozygous mutants. amy, amydala; cb, cerebellum; cc, corpus callosum; cx, cortex; fim, fimbria; hip, hippocampus; hy, hypothalamus; ic, inferior colliculus; med, medulla oblongata; pt, pretectum; sc, superior colliculus; th, thalamus. Scale bar: 500 µm.
(5.51 MB TIF)

Figure S5 The effect of chPlxdc2-Myc misexpression at HH stage 10–11 on the brain at later stages of development. a and b, external wholemount images of an experimental embryo collected 5 days post electroporation (HH stage 29), illustrating no gross morphological defect in brain size or shape. EGFP expression is still clearly visible on the experimental side of the brain (b). c, dorsal view of a HH stage 38 embryo brain, dissected 10 days post electroporation with chPlxdc2-Myc. cb, cerebellum; med, medulla oblongata; mes, mesencephalon; MHB, midbrain-hindbrain boundary; tec, optic tectum; tel, telencephalon. Scale bar: a, 1 mm; b, 0.5 mm; c, 2 mm.
(2.46 MB TIF)

Figure S6 In vivo knockdown of Plxdc2 by shRNAs targeted to the gene had no effect on neural tube thickness. Four shRNAs were designed as per Bron et al., 2004 and ligated into the pCA-β-EGFPm5-U6 vector. ShRNA's were tested in pairs in vitro by co-transfection with chPlxdc2-Myc. 1 µg of chPlxdc2-Myc and 1 µg shRNA plasmid (in total) were co-transfected into HEK293T cells at 60% confluency using Fugene HD Transfection Reagent (Roche). Cells were cultured for a further 18 hours before immunocytochemistry. For western blotting, protein was collected at 18 hours post transfection. a–f, Immunocytochemistry showing knockdown of chPlxdc2-Myc by the most efficient shRNA pair (shRNA1&3) (a–c). The empty pCA-β-EGFPm5-U6 plasmid caused no knockdown of chPlxdc2-Myc (d–f). g, Western blot confirmation of immunocytochemistry results showing that shRNA1&3, used in combination, caused the most dramatic knockdown of chPlxdc2-Myc (ii). i, shRNA1&2; ii, shRNA1&3; iii, shRNA1&4; iv, shRNA2&3; v, negative control of untransfected cells; vi, positive control of cells transfected with chPlxdc2-Myc and the pCA-β-EGFPm5-U6 plasmid; vii, shRNA2&4; viii, shRNA 3&4. h, Complete set of thickening ratios from multiple sections of individuals across in ovo electroporation experiments. Thickening ratios of specimens electroporated with chPlxdc2-Myc or Plxdc2-AP were significantly greater than those of control cases, 24 hours after electroporation (p≤0.0001 in both cases). As endogenous Plxdc2 expression occurs at the MHB of the chick, thickening ratios for shRNA experiments were calculated from OPT sections through this region. No significant effect on thickening ratio was observed 24 hours following electroporation of shRNA1&3 at the MHB. Thickening ratios in chPlxdc2-Myc and shRNA1&3 co-electroporated specimens did not differ significantly from control cases (p>0.05). n = number of sections analysed.
(7.82 MB TIF)

Table S1 Cell counts for BrdU-incorporation in ENC cultures treated with msPlxdc2-AP compared to those treated with AP alone. Cells for treatment with msPlxdc2-AP and AP alone were grown from the same starter culture. Duplicate coverslips per condition were used. Cell fields were photographed at five consistent locations on each coverslip. The total number of BrdU-negative cells (DAPI) and BrdU-positive cells per coverslip is shown. The proportion of BrdU-incorporation per culture is shown in bold text as the mean of 2 coverslips (normalised to total cell number).
(6.42 MB TIF)

Table S2 Percentage increase in BrdU-incorporation in ENC cultures treated with msPlxdc2-AP compared to those treated with AP alone.
(1.24 MB TIF)
