# Supplementary figures and images for: Plxdc2 Is a Mitogen for Neural Progenitors
Source: PLoS One. 2011 Jan 21;6(1):e14565. doi: 10.1371/journal.pone.0014565 (PMC3024984; doi:10.1371/journal.pone.0014565)

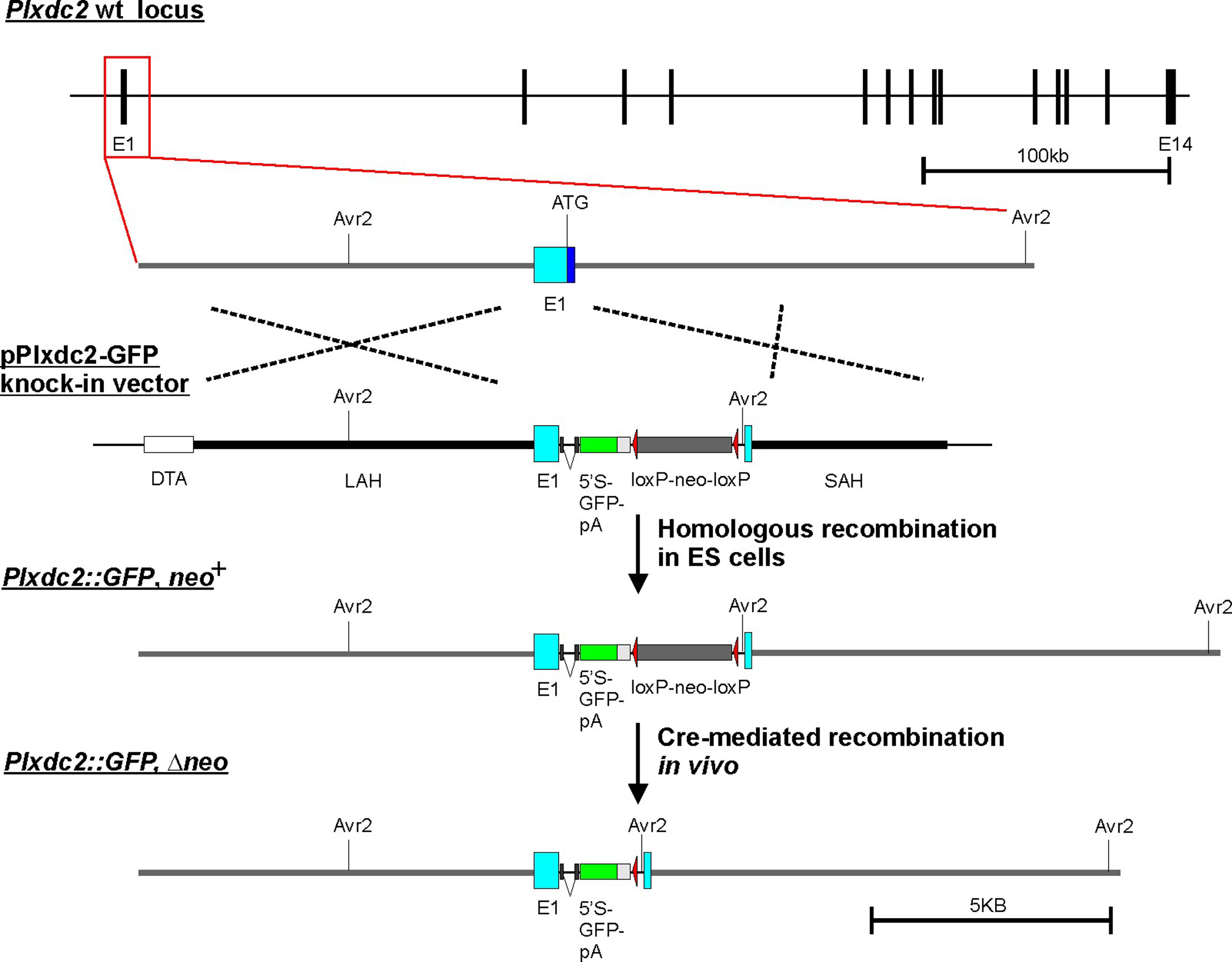

Supplement: Figure S1 — Design strategy for the creation of the Plxdc2GFP mouse line. The Plxdc2 gene is inactivated through replacement of the start codon & leader peptide by a 5S-EGFP-pA cassette following homologous recombination in ES cells. (9.84 MB TIF) [file pone.0014565.s001.tif]

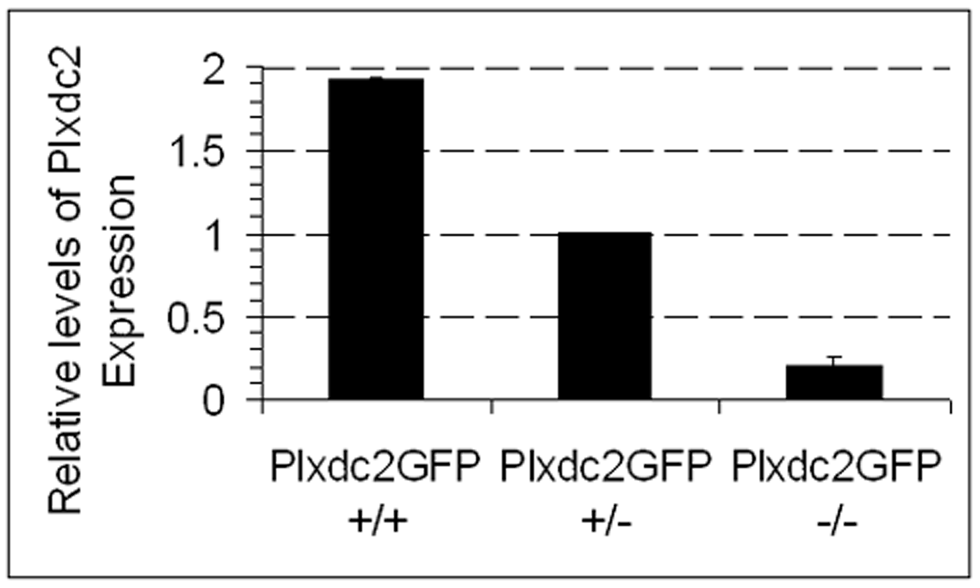

Supplement: Figure S2 — Examination of residual (exon 7) Plxdc2 transcript levels in Plxdc2GFP mice by realtime PCR. Samples for real time PCR were normalised to the mouse RPO gene. Plxdc2 transcript levels were significantly reduced in heterozygous Plxdc2GFP mice when compared to wildtype animals (independent T-test, p≤0.0001). Plxdc2 expression in heterozygous mutants was approximately half that evident in wildtype mice. Plxdc2 transcript levels were significantly reduced in homozygous Plxdc2GFP mice when compared to those in heterozygous animals (independent T-test, p≤0.0001). There was approximately a ten fold decrease in Plxdc2 transcript levels in the cerebella of Plxdc2GFP homozygous mutants, when compared to wildtype animals showing that in addition to abolishing normal protein translation and secretion (start codon and leader sequence removed), transcript levels from the locus are also greatly reduced by the genomic alteration. (1.73 MB TIF) [file pone.0014565.s002.tif]

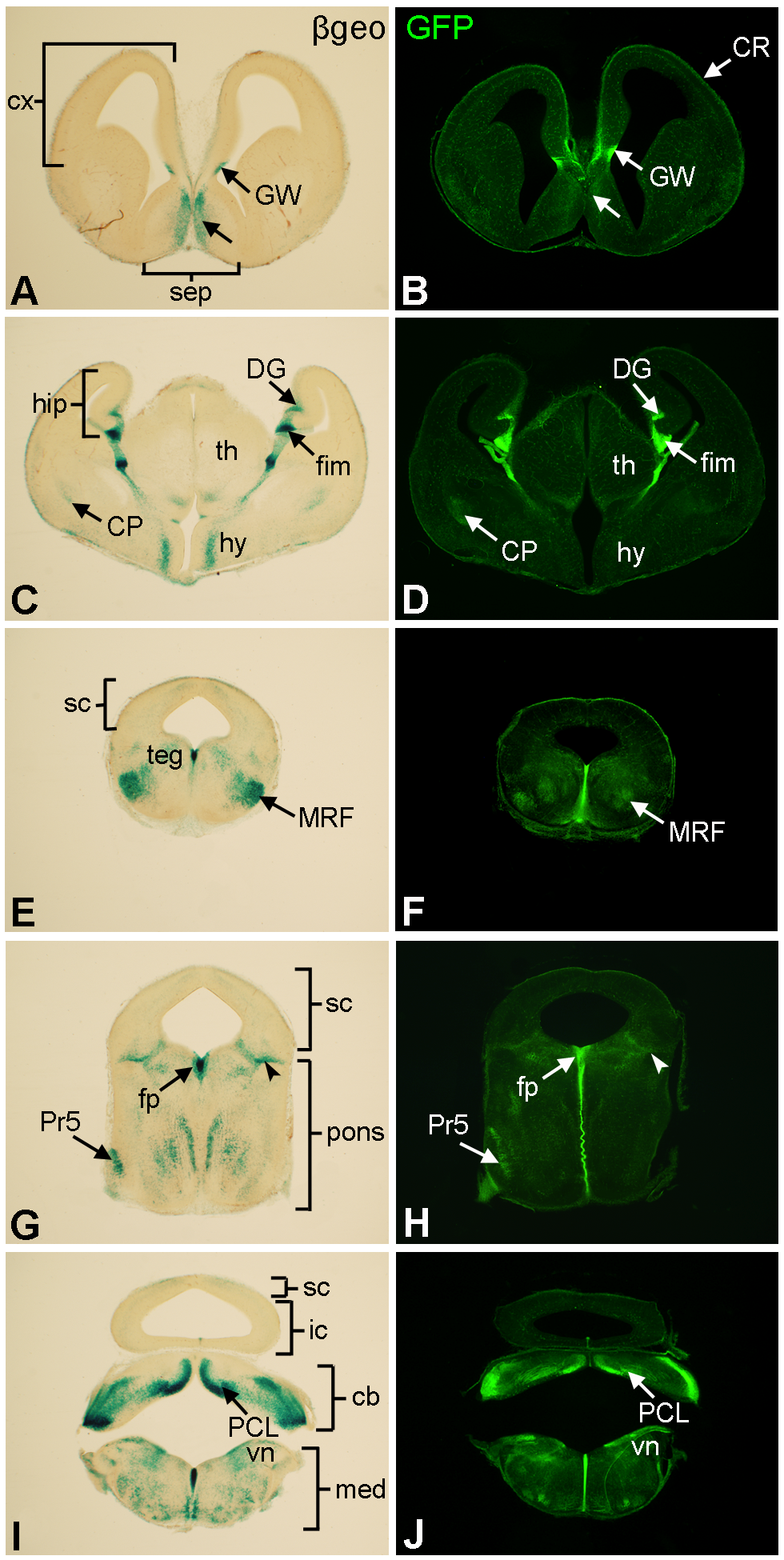

Supplement: Figure S3 — Plxdc2 expression in the E15.5 Plxdc2GFP mouse brain. GFP expression was compared to that of Plxdc2-βgeo in heterozygous PLAP secretory trap mice at the same stage of development. Plxdc2 expression in the Plxdc2GFP mouse line mirrored that in the PLAP secretory trap line in all areas of the E15.5 brain. Representative images through the brain are shown illustrating GFP expression in many regions of the E15.5 brain including the glial wedge (GW), fimbria (fim), dentate gyrus (DG), caudate putamen (CP), midbrain reticular formation (MRF), floor plate (fp), principle sensory trigeminal nucleus (Pr5), Purkinje cell layer (PCL) and vestibular nuclei (vn). a,c,e,g and i: coronal sections through the brain of a heterozygous Plxdc2 gene trap mouse illustrating Plxdc2-βgeo expression. b,d,f,h and j: corresponding coronal sections through the brain of a heterozygous Plxdc2GFP mouse illustrating GFP expression. arrow in a and b, Plxdc2 expression at the medial septum; arrowhead in g and h, clusters of Plxdc2 expression at the border region of the tectum and the pons; cb, cerebellum; cx, cortex; hip, hippocampus; hy, hypothalamus; ic, inferior colliculus; med, medulla oblongata; sc, superior colliculus; sep; septum; teg, tegmentum; th, thalamus. Scale bar:1 mm. (5.74 MB TIF) [file pone.0014565.s003.tif]

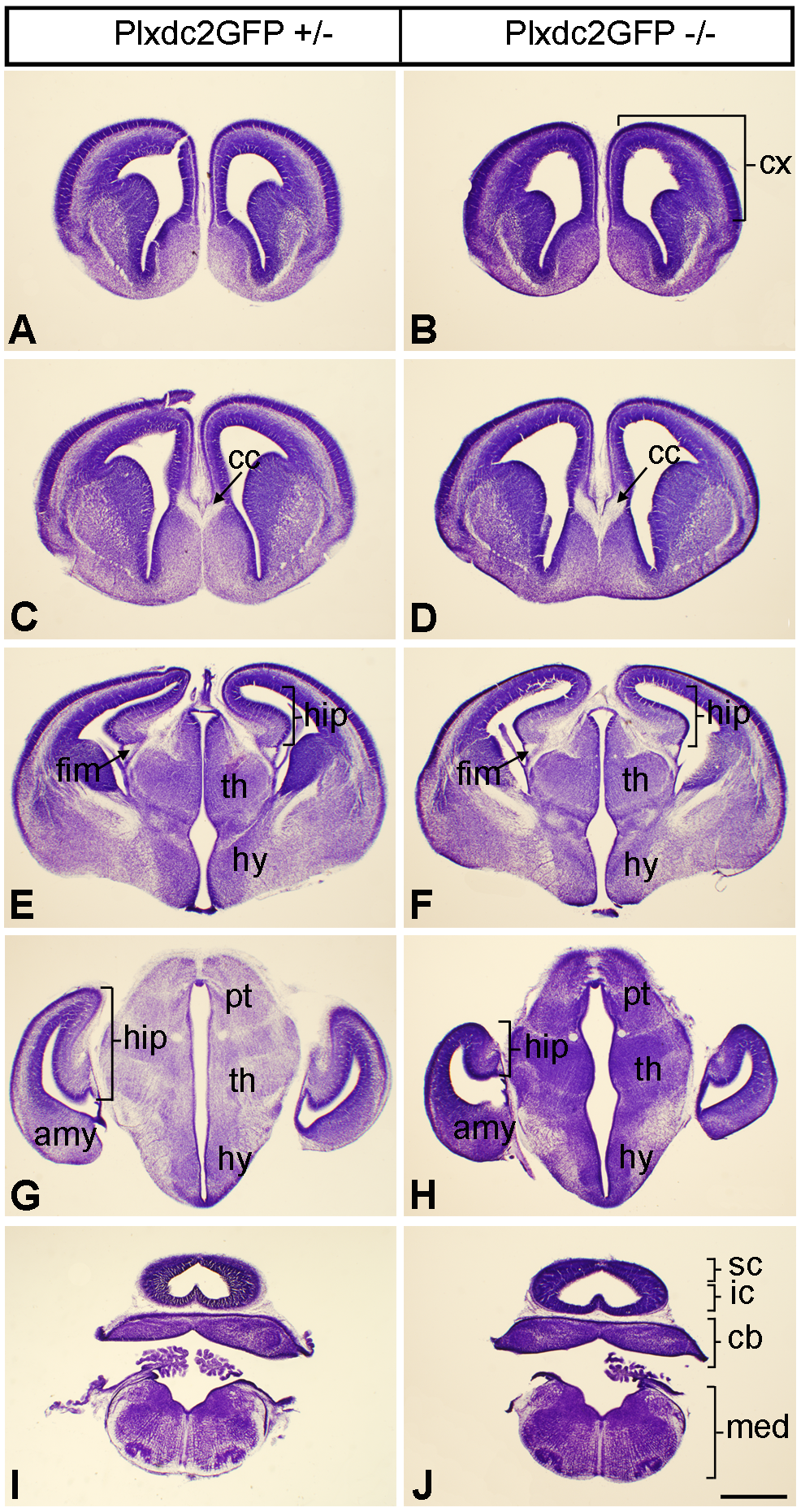

Supplement: Figure S4 — Cresyl violet staining of coronal sections through the brain of E15.5 Plxdc2GFP mice. No gross morphological phenotype was evident in Plxdc2GFP homozygous mutants. amy, amydala; cb, cerebellum; cc, corpus callosum; cx, cortex; fim, fimbria; hip, hippocampus; hy, hypothalamus; ic, inferior colliculus; med, medulla oblongata; pt, pretectum; sc, superior colliculus; th, thalamus. Scale bar: 500 µm. (5.51 MB TIF) [file pone.0014565.s004.tif]

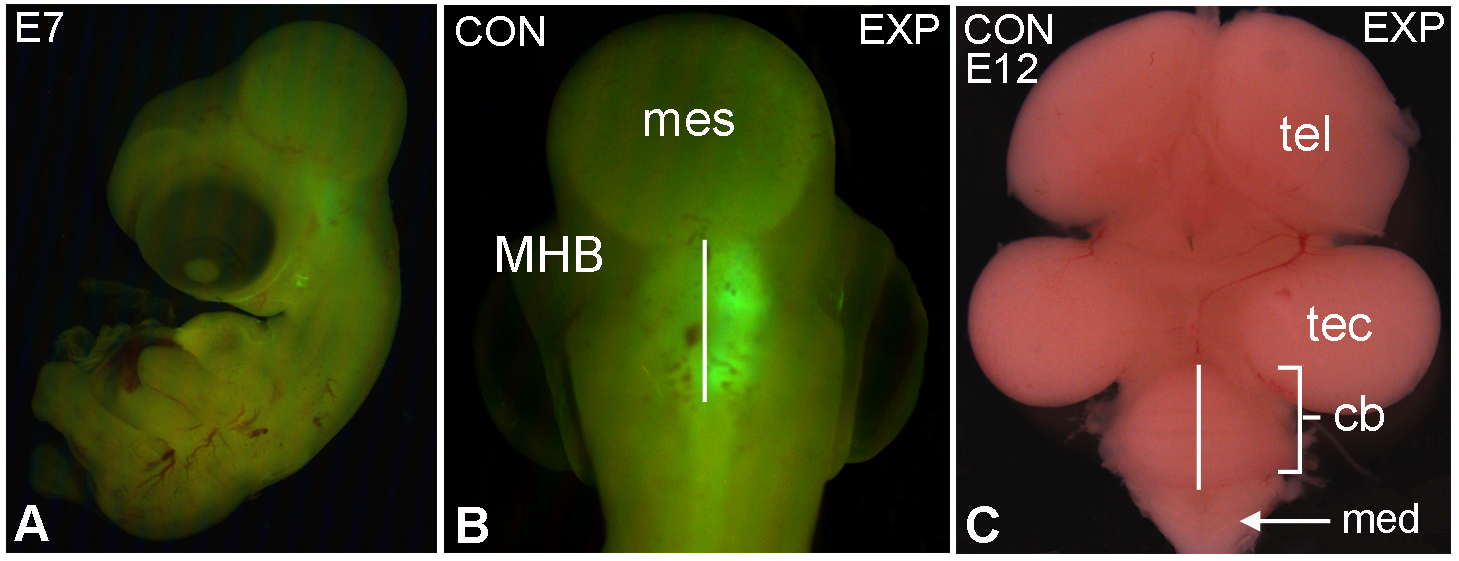

Supplement: Figure S5 — The effect of chPlxdc2-Myc misexpression at HH stage 10–11 on the brain at later stages of development. a and b, external wholemount images of an experimental embryo collected 5 days post electroporation (HH stage 29), illustrating no gross morphological defect in brain size or shape. EGFP expression is still clearly visible on the experimental side of the brain (b). c, dorsal view of a HH stage 38 embryo brain, dissected 10 days post electroporation with chPlxdc2-Myc. cb, cerebellum; med, medulla oblongata; mes, mesencephalon; MHB, midbrain-hindbrain boundary; tec, optic tectum; tel, telencephalon. Scale bar: a, 1 mm; b, 0.5 mm; c, 2 mm. (2.46 MB TIF) [file pone.0014565.s005.tif]

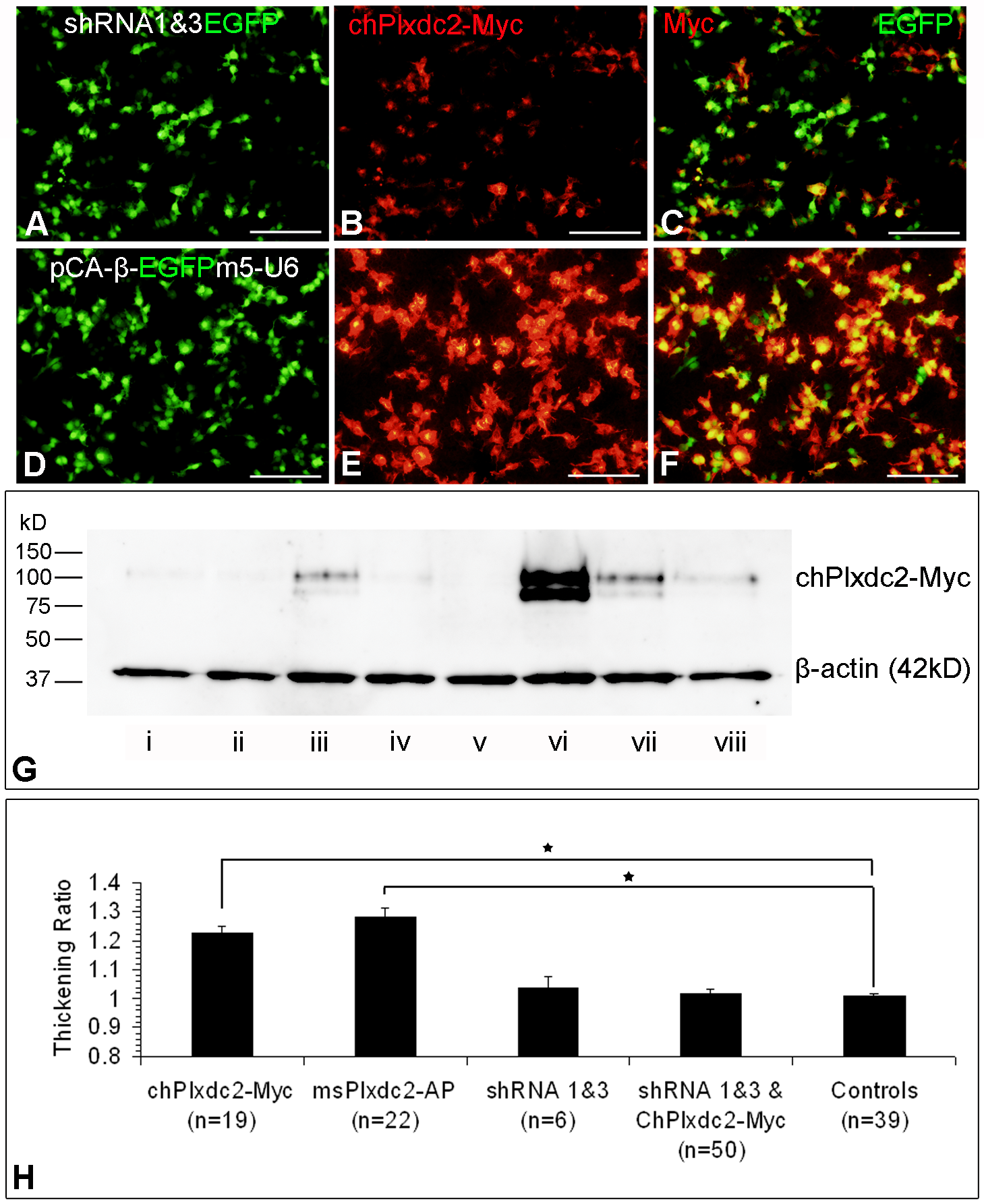

Supplement: Figure S6 — In vivo knockdown of Plxdc2 by shRNAs targeted to the gene had no effect on neural tube thickness. Four shRNAs were designed as per Bron et al., 2004 and ligated into the pCA-β-EGFPm5-U6 vector. ShRNA's were tested in pairs in vitro by co-transfection with chPlxdc2-Myc. 1 µg of chPlxdc2-Myc and 1 µg shRNA plasmid (in total) were co-transfected into HEK293T cells at 60% confluency using Fugene HD Transfection Reagent (Roche). Cells were cultured for a further 18 hours before immunocytochemistry. For western blotting, protein was collected at 18 hours post transfection. a–f, Immunocytochemistry showing knockdown of chPlxdc2-Myc by the most efficient shRNA pair (shRNA1&3) (a–c). The empty pCA-β-EGFPm5-U6 plasmid caused no knockdown of chPlxdc2-Myc (d–f). g, Western blot confirmation of immunocytochemistry results showing that shRNA1&3, used in combination, caused the most dramatic knockdown of chPlxdc2-Myc (ii). i, shRNA1&2; ii, shRNA1&3; iii, shRNA1&4; iv, shRNA2&3; v, negative control of untransfected cells; vi, positive control of cells transfected with chPlxdc2-Myc and the pCA-β-EGFPm5-U6 plasmid; vii, shRNA2&4; viii, shRNA 3&4. h, Complete set of thickening ratios from multiple sections of individuals across in ovo electroporation experiments. Thickening ratios of specimens electroporated with chPlxdc2-Myc or Plxdc2-AP were significantly greater than those of control cases, 24 hours after electroporation (p≤0.0001 in both cases). As endogenous Plxdc2 expression occurs at the MHB of the chick, thickening ratios for shRNA experiments were calculated from OPT sections through this region. No significant effect on thickening ratio was observed 24 hours following electroporation of shRNA1&3 at the MHB. Thickening ratios in chPlxdc2-Myc and shRNA1&3 co-electroporated specimens did not differ significantly from control cases (p>0.05). n = number of sections analysed. (7.82 MB TIF) [file pone.0014565.s006.tif]

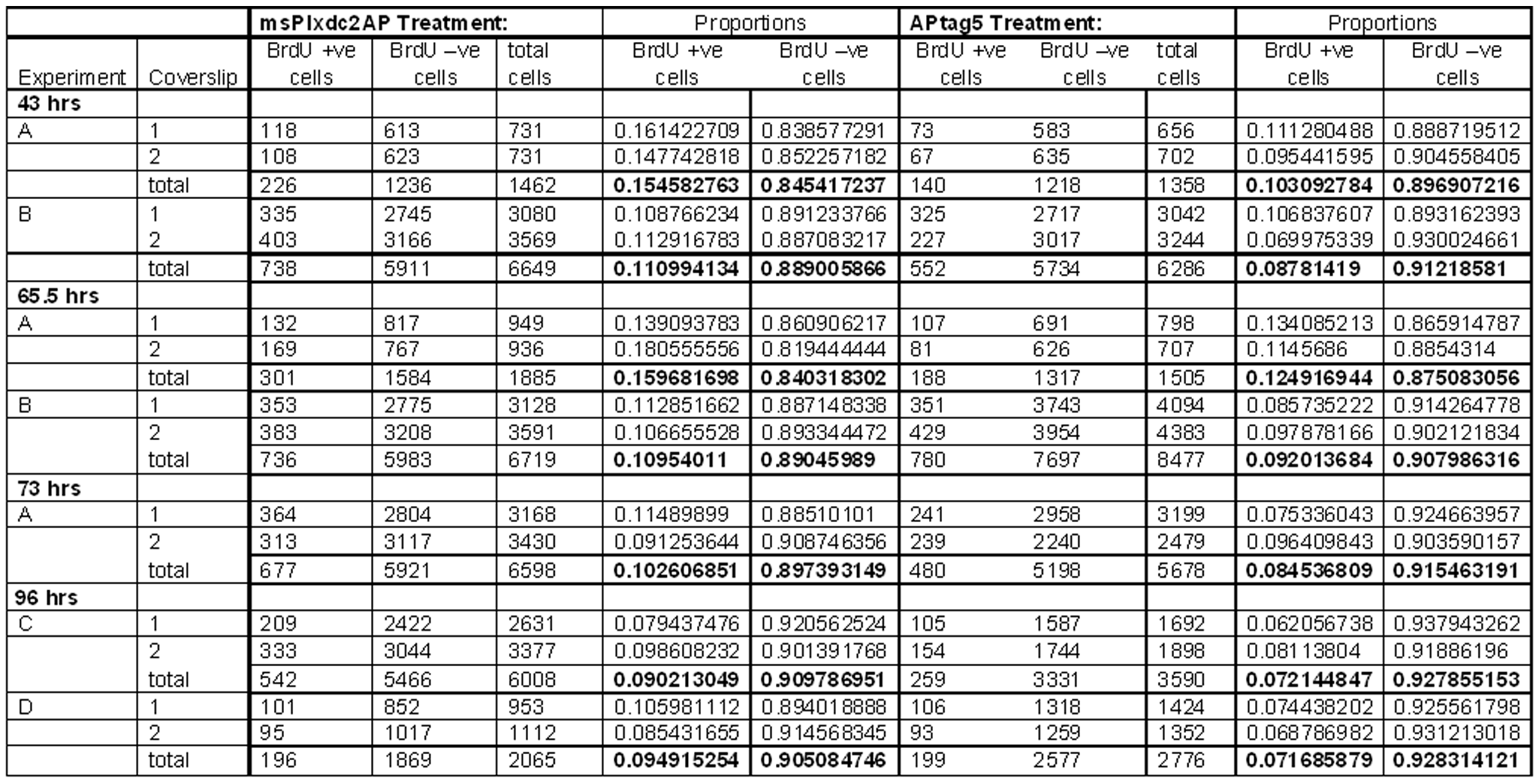

Supplement: Table S1 — Cell counts for BrdU-incorporation in ENC cultures treated with msPlxdc2-AP compared to those treated with AP alone. Cells for treatment with msPlxdc2-AP and AP alone were grown from the same starter culture. Duplicate coverslips per condition were used. Cell fields were photographed at five consistent locations on each coverslip. The total number of BrdU-negative cells (DAPI) and BrdU-positive cells per coverslip is shown. The proportion of BrdU-incorporation per culture is shown in bold text as the mean of 2 coverslips (normalised to total cell number). (6.42 MB TIF) [file pone.0014565.s007.tif]

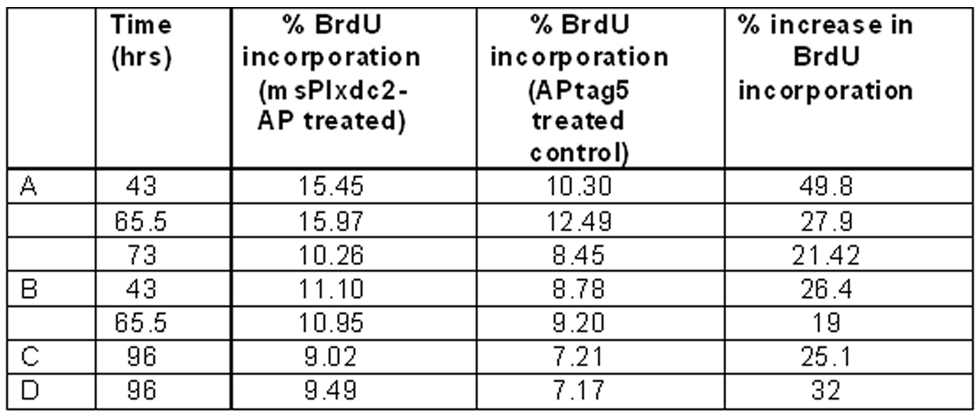

Supplement: Table S2 — Percentage increase in BrdU-incorporation in ENC cultures treated with msPlxdc2-AP compared to those treated with AP alone. (1.24 MB TIF) [file pone.0014565.s008.tif]
